# Blood coagulation protein fibrinogen promotes autoimmunity and demyelination via chemokine release and antigen presentation

**DOI:** 10.1038/ncomms9164

**Published:** 2015-09-10

**Authors:** Jae Kyu Ryu, Mark A. Petersen, Sara G. Murray, Kim M. Baeten, Anke Meyer-Franke, Justin P. Chan, Eirini Vagena, Catherine Bedard, Michael R. Machado, Pamela E. Rios Coronado, Thomas Prod'homme, Israel F. Charo, Hans Lassmann, Jay L. Degen, Scott S. Zamvil, Katerina Akassoglou

**Affiliations:** 1 Gladstone Institute of Neurological Disease, University of California, San Francisco, California 94158, USA; 2 Division of Neonatology, Department of Pediatrics, University of California San Francisco, San Francisco, California 94143, USA; 3 Department of Neurology, University of California San Francisco, San Francisco, California 94143, USA; 4 Program in Immunology, University of California San Francisco, San Francisco, California 94143, USA; 5 Gladstone Institute of Cardiovascular Disease, University of California, San Francisco, California 94158, USA; 6 Centre for Brain Research, Medical University of Vienna, Vienna A-1090, Austria; 7 Division of Experimental Hematology, Cincinnati Children's Hospital Research Foundation and University of Cincinnati College of Medicine, Cincinnati, Ohio 45229, USA

## Abstract

Autoimmunity and macrophage recruitment into the central nervous system (CNS) are critical determinants of neuroinflammatory diseases. However, the mechanisms that drive immunological responses targeted to the CNS remain largely unknown. Here we show that fibrinogen, a central blood coagulation protein deposited in the CNS after blood–brain barrier disruption, induces encephalitogenic adaptive immune responses and peripheral macrophage recruitment into the CNS leading to demyelination. Fibrinogen stimulates a unique transcriptional signature in CD11b^+^ antigen-presenting cells inducing the recruitment and local CNS activation of myelin antigen-specific Th1 cells. Fibrinogen depletion reduces Th1 cells in the multiple sclerosis model, experimental autoimmune encephalomyelitis. Major histocompatibility complex (MHC) II-dependent antigen presentation, CXCL10- and CCL2-mediated recruitment of T cells and macrophages, respectively, are required for fibrinogen-induced encephalomyelitis. Inhibition of the fibrinogen receptor CD11b/CD18 protects from all immune and neuropathologic effects. Our results show that the final product of the coagulation cascade is a key determinant of CNS autoimmunity.

## Abstract (web summary)

Autoimmune brain inflammation is associated with activation of macrophages and microglia. Here the authors show that fibrinogen induces encephalitogenic T-cell activation and macrophage recruitment to the central nervous system, and promotes demyelination in a mouse model of multiple sclerosis.

## Graphical abstract

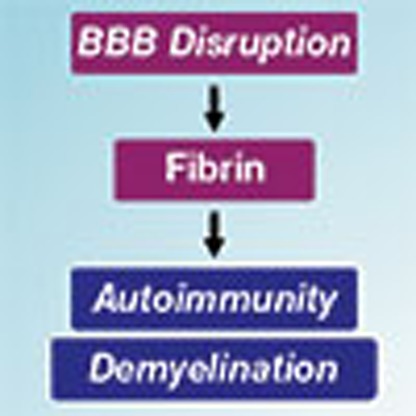

Disruption of the homeostatic balance between the vasculature and the brain is a sustained and often early feature of neurologic diseases and traumatic insults to the central nervous system (CNS). Understanding how blood–brain barrier (BBB) disruption instigates and amplifies immune and degenerative responses leading to brain pathology and loss of function would be instrumental in the design of novel treatments for neurologic diseases. Fibrinogen (coagulation factor I) is a major component in the blood that upon BBB disruption enters the CNS and is deposited as insoluble fibrin^1^. Although soluble fibrinogen in the bloodstream is not proinflammatory, activation of the coagulation cascade results in the formation of fibrin associated with exposure of cryptic epitopes that transform fibrinogen from a blood factor to a potent activator of innate immunity^1^. In multiple sclerosis (MS) and experimental autoimmune encephalomyelitis (EAE), BBB disruption and fibrin deposition are detected in the white matter along with microglial activation before T-cell infiltration and the onset of demyelination^2^,^3^,^4^,^5^,^6^. Indeed, increased coagulation activity leading to fibrin formation occurs early in neuroinflammation before demyelination^4^, and is highly upregulated in active MS plaques^7^. Moreover, injection of soluble fibrinogen in the healthy brain results in fibrin formation^5^. Fibrin deposition is abundant not only in early but also active and chronic MS lesions, and correlates with demyelination and T-cell infiltration^3^,^8^,^9^,^10^. Genetic or pharmacologic depletion of fibrinogen decreases microglial activation and axonal damage and attenuates neurologic signs in EAE^5^,^7^,^11^,^12^,^13^ and other MS models^14^,^15^. While studies in EAE mice support a role for fibrin in the activation of microglia in myelinated areas, its role in the initiation and propagation of myelin-targeted adaptive immune responses is unknown. Moreover, despite the vast literature on BBB disruption and activation of the coagulation cascade in brain diseases, there is currently no model of neuroinflammation induced by a coagulation factor.

Here we developed a model of coagulation-driven demyelination to directly assess the role of BBB disruption and fibrin in the induction of CNS autoimmunity and demyelination. Surprisingly, introduction of fibrinogen into the healthy CNS was sufficient to induce activation of adaptive immunity targeted to CNS myelin antigens leading to demyelination. The effect of fibrinogen as an initiator of CNS autoimmunity was first substantiated *in vivo*, where a single stereotactic injection of fibrinogen in the corpus callosum induced recruitment and local differentiation of myelin antigen-specific Th1 cells leading to demyelination. In accordance, endogenous fibrinogen was required for T-helper 1 (Th1) cell activation in EAE. Microarray analysis in cell autonomous systems of antigen-presenting cells (APCs) uncovered a unique transcriptional signature for fibrin that links the coagulation cascade with M1-type activation of APCs associated with induction of antigen presentation and release of leukocyte recruiting chemokines. Rescue experiments to genetically deplete the fibrin-specific immune responses in APCs demonstrated that secretion of the monocyte chemoattractant protein-1 (MCP-1/CCL2) and the C-X-C motif ligand-10 (CXCL10), together with MHC II-dependent antigen presentation, are essential molecular mediators for fibrin-induced autoimmune responses in the CNS. Furthermore, we showed that fibrin induces CNS autoimmune responses not via its prohaemostatic functions, but due to CD11b/CD18 receptor activation in APCs. Our results show for the first time that a component of the coagulation cascade induces autoimmunity, attributes the CNS effects of coagulation to the proinflammatory effects of fibrin and introduces fibrinogen-induced encephalomyelitis (FIE) as a novel coagulation-driven, autoimmune-mediated model for the study of novel mechanisms and therapies for brain inflammation.

## Results

### Fibrinogen in the CNS induces inflammatory demyelination

**Figure 1:**
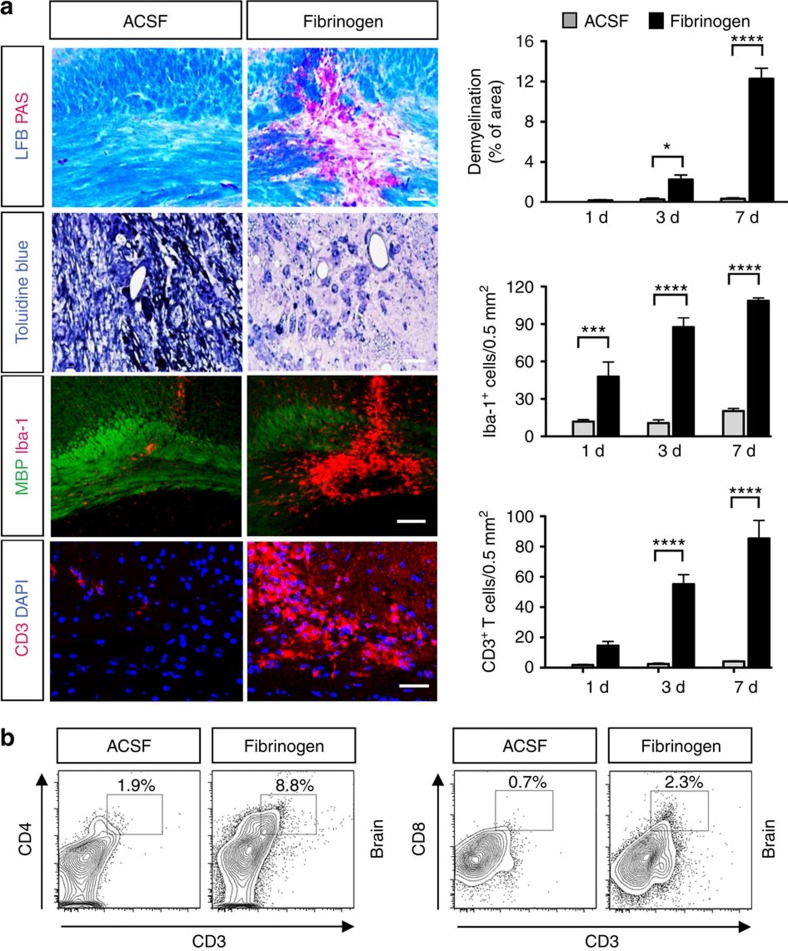
Induction of T-cell recruitment and inflammatory demyelination by a single fibrinogen injection in the CNS. (**a**) Demyelination (LFB/PAS and toluidine blue), microglial activation and demyelination (MBP/Iba-1), and T-cell infiltration (CD3) in the corpus callosum of mice injected with fibrinogen compared with ACSF control. Scale bar, 100 μm (top panel); 10 μm (second panel); 100 μm (third panel); 80 μm (bottom panel). Representative histological sections from day 7 after injection are shown. Data are presented as mean±s.e.m. (*n*=5–6 mice per time point). *P<0.05, ***P<0.001, ****P<0.0001 (two-way ANOVA and Bonferroni's multiple comparisons test). (**b**) FACS analysis of T cells isolated from the brain (corpus callosum) 7 days after fibrinogen or ACSF injection stained with CD3, CD4 and CD8 (*n*=3 independent experiments; each experiment generated from pooled brain cells from *n*=3–4 mice). ANOVA, analysis of variance; d, days; FACS, fluorescence-activated cell sorting; LFB, Luxol fast blue.

To determine whether fibrinogen extravasation is sufficient to induce CNS pathology *in vivo*, we stereotactically injected fibrinogen at its physiological plasma concentration into the corpus callosum, the largest CNS white matter tract associated with disability in MS^16^. Surprisingly, this single injection of fibrinogen spontaneously induced local demyelination within 7 days (Fig. 1a). Microglial activation was rapidly induced 1 day after fibrinogen injection and preceded demyelination (Fig. 1a). Activation of innate immunity regulates autoimmune responses including T-cell recruitment and differentiation^17^,^18^. Fibrinogen injection induced infiltration of both CD4^+^ and CD8^+^ T-cell populations in the CNS, but not in the spleen (Fig. 1a,b; Supplementary Fig. 1). T-cell infiltration occurred as early as 1 day after injection and markedly increased 3 and 7 days after injection (Fig. 1a), thus also preceding demyelination. Injection of fibrinogen in the spinal cord ventral column also induced inflammatory demyelination (Supplementary Fig. 2). Neuropathologic alterations were not observed after injection of control artificial cerebrospinal fluid (ACSF) in either the brain or spinal cord (Fig. 1; Supplementary Fig. 2). Although microglia show a diffuse response in the cortex along the needle track 1 day after ACSF injection, this response resolves and no alterations are observed either in the cortex or the corpus callosum (Fig. 1). While wild-type (WT) plasma induced demyelination similar to fibrinogen, injection of plasma from fibrinogen-deficient (*Fib*^−/−^) mice^19^, which contains all plasma proteins except fibrinogen, led to an 82% reduction in demyelination compared with WT plasma (Supplementary Fig. 3). Injection of the plasma proteins albumin or high-molecular-weight kininogen had no effect (Supplementary Fig. 3).

Activation of the coagulation cascade converts fibrinogen to proinflammatory fibrin primarily due to exposure of a cryptic epitope in the fibrinogen γ chain (γ_377–395_), which binds to CD11b/CD18 integrin (Mac-1, complement receptor 3, α_M_β_2_)^20^,^21^. The interaction of fibrin with CD11b/CD18 is genetically targeted in *Fib*γ^*390–396A*^ mice, in which fibrinogen has been mutated to lack the CD11b/CD18-binding motif, but retains normal clotting function^22^. Injection of plasma derived from *Fib*γ^*390–396A*^ mice resulted in a 70% reduction in demyelination compared with WT plasma (Supplementary Fig. 3), suggesting that the interaction of fibrin with CD11b/CD18 is required for the induction of demyelination. Since a portion of fibrinogen in the plasma is known to bind extracellular matrix proteins and growth factors, we also produced and tested recombinant fibrinogen, which is clottable and hydrodynamically indistinguishable from plasma fibrinogen, with the exception that it did not carry other plasma-derived factors^23^. Similar to plasma fibrinogen, recombinant fibrinogen also induced demyelination and microglial activation (Supplementary Fig. 4). These results suggest that fibrinogen is a major component in the plasma that in the healthy CNS white matter triggers T-cell recruitment and demyelination even in the absence of pre-existing inflammatory or myelin abnormalities.

### Fibrinogen induces M1-type activation of APCs

**Figure 2:**
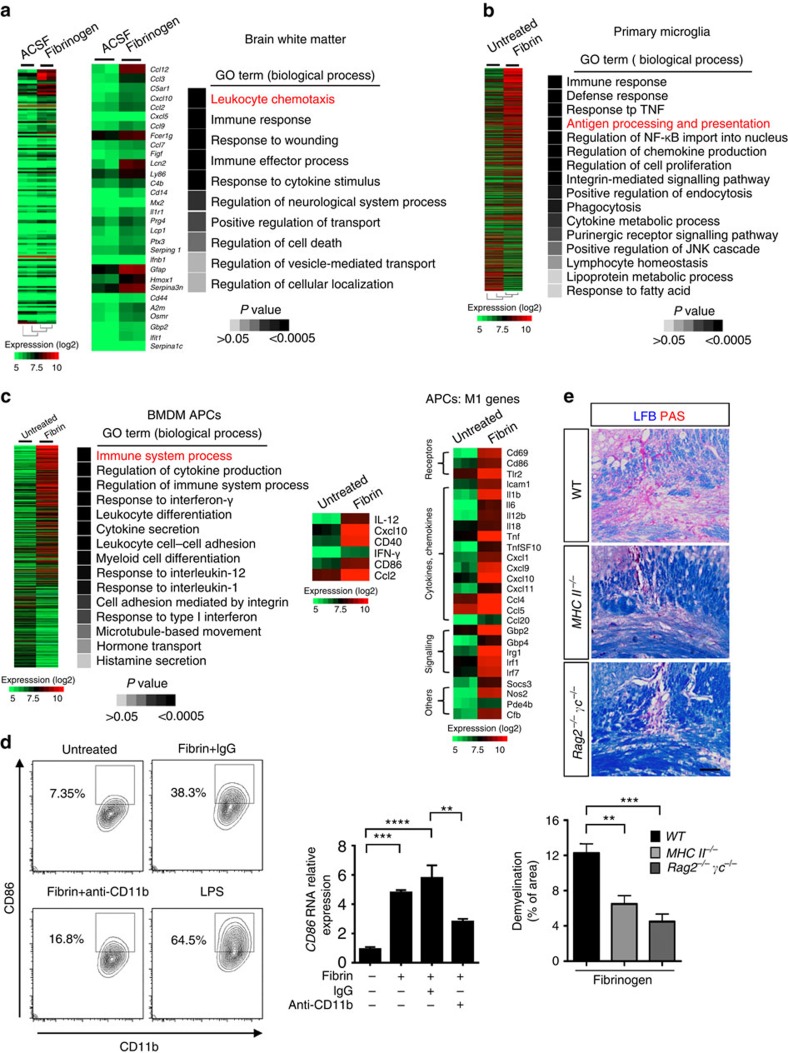
Adaptive immunity and antigen presentation are required for fibrinogen-induced demyelination. (**a**) Affymetrix microarray gene expression analysis and enrichment of gene ontology (GO) of fibrinogen-injected or ACSF-injected corpus callosum at 12 h post injection. Heatmaps of the 142 genes with ≥1.5 × change in expression between ACSF and fibrinogen. (**b**) Affymetrix microarray gene expression analysis of fibrin-stimulated rat primary microglia at 6 h *in vitro*. Heatmap and GO analysis of gene expression profiles of 1,342 genes with ≥1.5 × change in expression between unstimulated and fibrin treatment. (**c**) Affymetrix microarray gene expression analysis of fibrin-stimulated mouse APCs at 6 h *in vitro*. Heatmap and GO analysis of gene expression profiles of key genes with ≥1.5 × change in expression (left). Heatmap of the M1-related genes with ≥2.0 × change in expression after fibrin stimulation (right). (**d**) FACS analysis of CD86 expression in APCs after fibrin stimulation. LPS was used as positive control. Anti-CD11b antibody treatment reduces CD86 expression in APCs. Real-time PCR analysis of *CD86* gene expression in BMDMs after fibrin stimulation treated with anti-CD11b or IgG isotype control antibody. Data are presented as mean±s.e.m. (*n*=4 independent experiments; right). **P<0.01, ***P<0.001, ****P<0.0001 (one-way ANOVA). (**e**) Demyelination (LFB/PAS) in the corpus callosum of WT, *MHC II*^−/−^ or *RAG2*^−/−^γ*c*^−/−^ mice 7 days after fibrinogen injection. Representative images are shown. Scale bar, 100 μm. Data are presented as mean±s.e.m. (*n*=6 mice per group). **P<0.01, ***P<0.001 (one-way ANOVA and Bonferroni's multiple comparisons test). ANOVA, analysis of variance; FACS, fluorescence-activated cell sorting; LFB, Luxol fast blue.

Genome-wide microarray analysis either in the corpus callosum after fibrinogen injection or in cell autonomous systems of fibrin-stimulated microglia or bone marrow-derived macrophages (BMDMs) revealed a unique fibrin transcriptional signature enriched in genes regulating immune responses, particularly those required to induce activation of T cells by APCs^24^, such as *MHC II, CD86* and *IL-12*, and recruitment of T cells and peripheral macrophage into the CNS^25^,^26^,^27^, such as *CXCL10* and *CCL2* (Fig. 2a–d; Supplementary Tables 1–3). Other immune response genes, such as complement components, lipocalin and proteins involved in iron binding and oxidative stress, were also increased. Fibrin induced M1-type activation and induction of antigen-presenting genes in both primary microglia and BMDMs (Fig. 2c; Supplementary Fig. 5). Consistent with these findings, protein and gene expression of MHC class II and CD86 were also induced in fibrin-exposed BMDMs, and were inhibited by anti-CD11b treatment (Fig. 2d; Supplementary Fig. 6). Lipopolysaccharide (LPS) was used as a positive control (Fig. 2d; Supplementary Fig. 6). In agreement, fibrinogen injection in the corpus callosum induced MHC class II and CXCL10 in Iba-1^+^ cells (Supplementary Fig. 7a,b). Similar to fibrinogen, injection of WT plasma in the corpus callosum induced *CXCL10* expression, while plasma derived from *Fib*^−/−^ or *Fib*γ^*390–396A*^ mice showed a significant reduction (Supplementary Fig. 7c). Strikingly, *MHC II*^−/−^ (ref. 28), *Cxcl10*^−/−^ (ref. 29) and *RAG2*^−/−^*γc*^−/−^ (ref. 30) mice, which lack T, B and natural killer cells, had a marked reduction in demyelination after fibrinogen injection compared with WT mice (Fig. 2e). *Cxcl10*^−/−^ mice also had 54% less T-cell infiltration than WT mice after fibrinogen injection (Supplementary Fig. 8). Overall, these results suggest that fibrin is a potent activator of APCs that triggers demyelination via T-cell recruitment into the CNS.

### Fibrinogen promotes recruitment of encephalitogenic T cells

**Figure 3:**
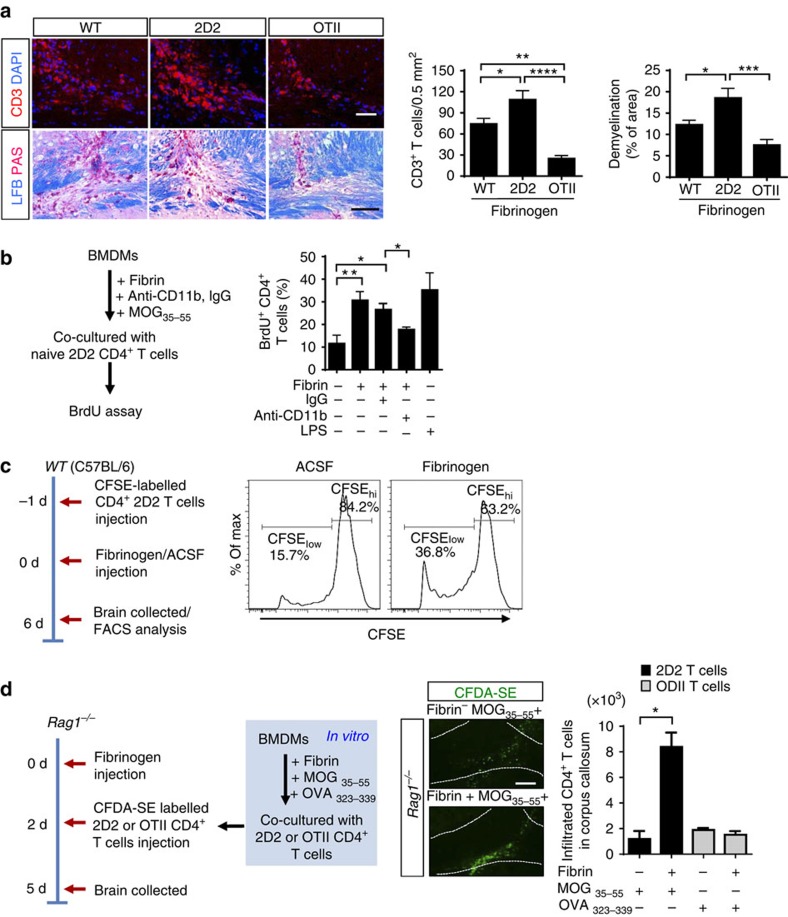
Extravascular fibrinogen drives myelin-specific T cells into the CNS. (**a**) T-cell infiltration (CD3) and demyelination (LFB/PAS) in the corpus callosum of WT, 2D2 and OT-II mice 7 days after fibrinogen injection. Scale bars, 100 μm (top panel); 200 μm (bottom panel). Quantification of CD3^+^ T cells and demyelination at 7 days after fibrinogen injection. Data are presented as mean±s.e.m. (*n*=6 mice per group). *P<0.05, **P<0.01, ***P<0.001, ****P<0.0001 by one-way ANOVA and Bonferroni's multiple comparisons test. (**b**) BMDMs treated with fibrin alone or in the presence of anti-CD11b or isotype IgG antibody control were co-cultured with naive 2D2 CD4^+^ T cells. BrdU proliferation assay in response to MOG_35–55_ peptide after fibrin stimulation and anti-CD11b treatment. LPS was used as positive control. Data are presented as mean±s.e.m. (*n*=3–4 independent experiments, *P<0.05, **P<0.01 by one-way ANOVA and Bonferroni's multiple comparisons test). (**c**) WT recipient mice received CFSE-labelled CD4^+^CD62L^+^ 2D2 T cells (0 d) and 1 day later fibrinogen or ACSF were injected in the corpus callosum. CFSE-labelled 2D2 T cells were isolated from the brains of fibrinogen- or ACSF-injected mice 6 days later and analysed by FACS. FACS plots and quantification showing CFSE dilution (CFSE_low_) indicate proliferation of 2D2 T cells in fibrinogen-injected brain. Data are representative of two independent experiments, each from pooled brain cells from *n*=4 mice. (**d**) Naive CD4^+^ 2D2 or control OT-II T cells were co-cultured with BMDMs treated with the indicated peptides, MOG_35–55_ or OVA_323–339_ alone or peptide in the presence of fibrin. CFDA-SE-labelled 2D2 or OT-II T cells were transferred into *Rag1*^−/−^ mice injected with fibrinogen in the corpus callosum. CFDA-SE^+^ T cells (green) in the corpus callosum (dashed line) of *Rag1*^−/−^ mice. Scale bar, 100 μm. Quantification shows increased 2D2 T cells after co-culture with BMDMs treated with both fibrin and MOG_35–55_, compared with fibrin and OVA_323–339_ or MOG_35–55_ alone. Data are presented as mean±s.e.m. (*n*=4 mice per group, *P<0.05 by non-parametric Mann–Whitney *U*-test). ANOVA, analysis of variance; d, days; FACS, fluorescence-activated cell sorting; LFB, Luxol fast blue.

To examine whether fibrinogen could induce T-cell responses against myelin antigens *in vivo*, we tested its effects in 2D2 transgenic mice constitutively expressing T-cell antigen receptors (TCRs) specific for myelin oligodendrocyte glycoprotein (MOG)^31^. After fibrinogen injection in the corpus callosum, T-cell infiltration and demyelination in 2D2 mice were 46% and 51% higher, respectively, compared with WT mice (Fig. 3a). In contrast, in control OT-II transgenic mice constitutively expressing TCRs specific for ovalbumin (OVA), an antigen not present in the CNS^32^, T-cell infiltration after fibrinogen injection was 77% and 66% less than in 2D2 or WT mice, respectively (Fig. 3a). Consistent with this result, demyelination was also significantly reduced in OT-II mice (Fig. 3a). Fibrin-treated BMDMs significantly increased the proliferation of MOG_35–55_-treated CD4^+^2D2 T cells in a CD11b-dependent manner, further suggesting that fibrin-induced activation of APCs promotes myelin-specific T-cell activation (Fig. 3b). LPS was used as a positive control (Fig. 3b).

### Fibrinogen promotes CNS expansion of encephalitogenic T cells

In addition to macrophages, microglia contribute to antigen presentation and T-cell activation during CNS autoimmune diseases^33^. This prompted us to investigate whether fibrin-induced microglial activation also influenced the activation of encephalitogenic T cells. Co-culture of microglia with naive 2D2 T cells in the presence of MOG peptide increased antigen-specific cell proliferation (Supplementary Fig. 9). Importantly, fibrin-stimulated microglia were more efficient APC for encephalitogenic T-cell proliferation than unstimulated microglia (Supplementary Fig. 9). On the basis of these findings, we asked whether fibrin induced activation of encephalitogenic T cells in the CNS. We labelled naive MOG-specific 2D2 T cells with carboxyfluorescein diacetate succinimidyl ester (CFSE) to monitor lymphocyte proliferation *in vivo*^34^,^35^. CFSE-labelled 2D2 T cells were adoptively transferred into fibrinogen- or control ACSF-injected WT mice and donor T cells were reisolated from the brain at 6 days after the transfer (Fig. 3c). MOG-specific 2D2 T cells proliferated in the brains of fibrinogen-injected recipients, compared with ACSF-injected controls (Fig. 3c), suggesting local T-cell activation. We next examined whether fibrin-induced activation of APCs promotes encephalitogenic T-cell trafficking into the CNS. Naive 2D2 or control OT-II T cells were co-cultured with BMDMs in the presence of MOG_35–55_ or OVA_323–339_, respectively. We adoptively transferred stimulated 2D2 or OT-II T cells into *Rag1*^−/−^ recipients that had been injected with fibrinogen and assessed migrated T cells in the corpus callosum after 3 days. Accumulation of 2D2 T cells in the corpus callosum was observed when donor 2D2 T cells were stimulated with their cognate antigen in the presence of fibrin-treated BMDMs, compared with only MOG_35–55_-treated cells (Fig. 3d). In contrast, fibrin-stimulated BMDMs did not promote the accumulation of OT-II T cells in the corpus callosum of *Rag1*^−/−^ recipients that had been injected with fibrinogen (Fig. 3d). These results suggest that fibrin signalling via CD11b/CD18 primes APCs, which facilitates the local expansion of antigen-specific encephalitogenic T cells within the CNS.

### Fibrinogen activates endogenous myelin antigen-specific T cells

**Figure 4:**
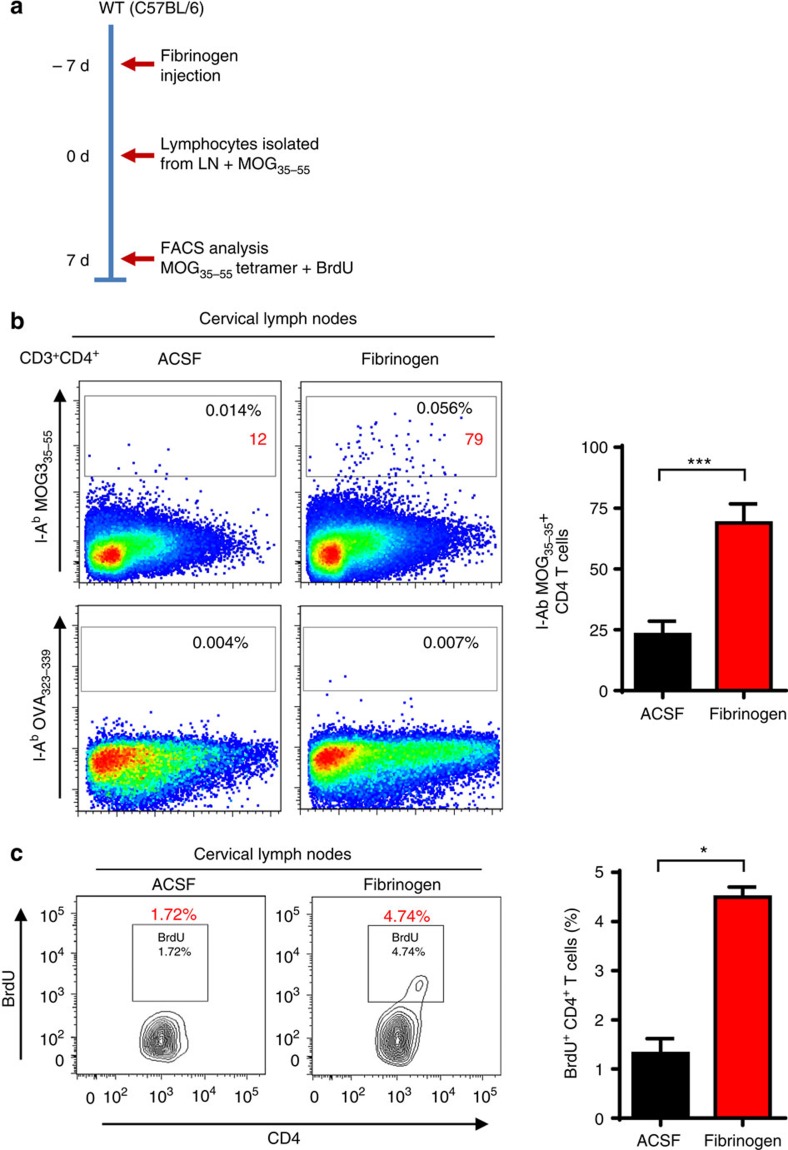
Fibrinogen drives accumulation of myelin antigen-specific T cells. (**a**) Experimental design diagram: C57BL/6 mice were stereotaxically injected with fibrinogen or ACSF in the corpus callosum. Seven days after injection, lymphocytes were prepared from draining lymph nodes and stimulated with MOG_35–55_ for 7 d. I-A^b^ MOG_35–55_ tetramer was used to detect myelin-specific CD4^+^ T cells in fibrinogen-injected WT mice. I-A^b^ OVA_323–339_ tetramer was used as a negative control for MOG_35–55_ tetramer staining. (**b**) Flow cytometry analysis of I-A^b^ MOG_35–55_ tetramer stained CD4^+^ T cells 7 days after MOG_35–55_ stimulation. No tetramer-positive cells were detected with I-A^b^ OVA_323–339_ tetramer. Graph shown number of I-A^b^ MOG_35–55_ tetramer stained CD4^+^ T cells. Data are presented as mean±s.e.m., *n*=8–9, with each sample being pooled from 2–3 mice from three independent experiments. ***P<0.001 (non-parametric Mann–Whitney *U*-test). (**c**) Proliferation analysis of BrdU incorporation in CD4^+^ T cells of ACSF- and fibrinogen-injected mice, stimulated with MOG_35–55_ for 7 days. Data are presented as mean±s.e.m. (four independent experiments with pooled cells from 2–3 mice per experiment for ACSF and fibrinogen), *P<0.05 (non-parametric Mann–Whitney *U*-test). d, days.

Local CNS activation of antigen-specific T cells in the brain drives autoimmune reactions^18^,^36^. Using the I-A^b^ MOG_35–55_ tetramer to detect in situ myelin-specific CD4^+^ T cells^37^,^38^, we examined whether fibrinogen entry in the CNS induces the local activation of myelin antigen-specific T cells. After 7 days post injection, isolated lymphocytes from draining lymph nodes were cultured with MOG_35–55_ peptide (Fig. 4a). *Ex vivo* staining with MOG_35–55_ tetramer of cells from draining lymph nodes showed a small but detectable fraction of tetramer-positive CD4 T cells only in fibrinogen-injected, but not in ACSF-injected mice. No tetramer-positive cells were detected on incubation with control I-A^b^ OVA_323–339_ tetramer (Fig. 4b), suggesting that fibrinogen induced myelin antigen-specific T-cell activation. Interestingly, only CD4^+^ T cells isolated from the cervical lymph nodes of fibrinogen-injected, but not ACSF-injected mice showed increased bromodeoxyuridine (BrdU) incorporation on MOG_35–55_ stimulation (Fig. 4c). To examine T-cell responses in the SJL/J mouse strain, lymphocytes were isolated from draining lymph nodes of fibrinogen-injected SJL/J mice and cultured with different proteolipid protein (PLP) peptides, including PLP_139–151_ and PLP_178–191_, which are immunodominant epitopes in the SJL/J mouse strain. CD4^+^ T cells of fibrinogen-injected SJL/J mice did not show significant proliferation in response to PLP_139–151_ or to ACSF (Supplementary Fig. 10), suggesting that perhaps isolation and expansion of a higher number of T cells from the draining lymph node and the CNS would be required to study responses to other myelin antigens after induction of FIE in other strains. In our study, MOG_35–55_ tetramer binding (Fig. 4b) and MOG_35–55_-induced BrdU incorporation (Fig. 4c) demonstrate the presence of functional autoreactive myelin antigen-specific CD4^+^ T cells in mice with fibrinogen-induced inflammatory demyelination.

### Fibrin induces Th1-cell differentiation via CD11b/CD18

**Figure 5:**
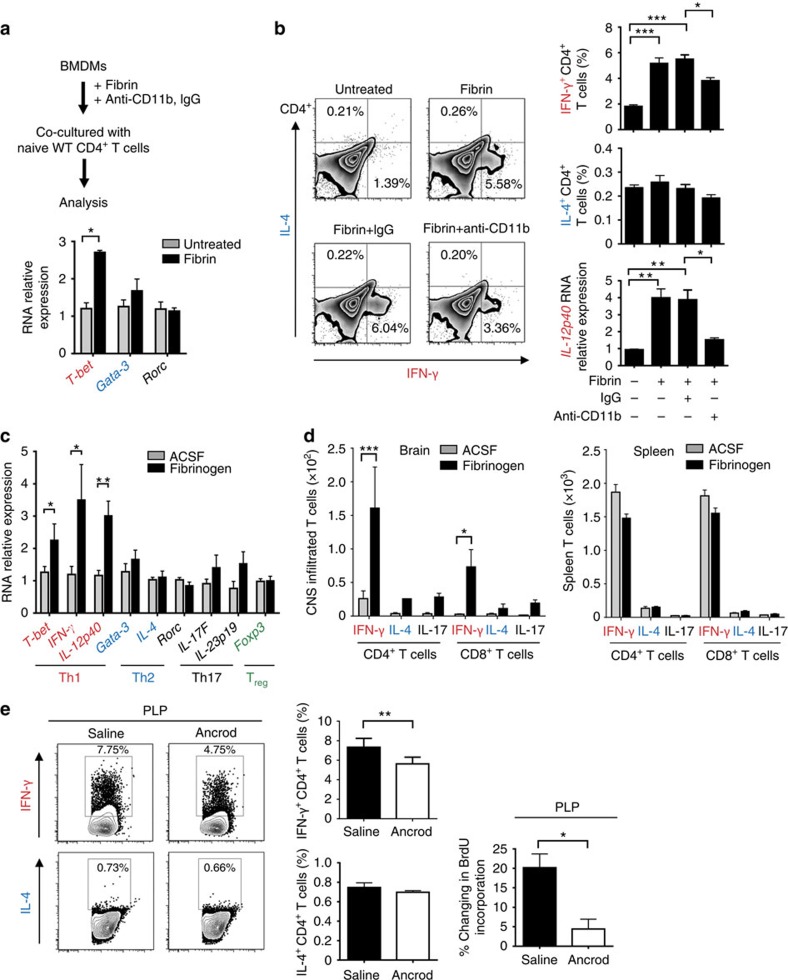
Fibrin induces activation of innate immunity via CD11b/CD18 to induce Th1-cell differentiation. (**a**) BMDMs treated with fibrin or in the presence of anti-CD11b or isotype IgG antibody control were co-cultured with naive WT CD4^+^ T cells and analysed for gene expression or by FACS. Gene expression analysis of transcriptional factors indicative of Th1, Th2, Th17 and T_reg_ cells in CD4^+^ T cells co-cultured with fibrin-stimulated BMDMs. Data are presented as mean±s.e.m. (*n*=4 independent experiments, *P<0.05 by non-parametric Mann–Whitney *U*-test). (**b**) Gated percentage of IFN-γ- or IL-4-expressing CD4^+^ T cells after co-culture with fibrin-stimulated BMDMs in the presence of rat IgG isotype control antibody or anti-CD11b antibody. Data are presented as mean±s.e.m. (*n*=3 independent experiments, *P<0.05, **P<0.01, ***P<0.001 by one-way ANOVA and Bonferroni's multiple comparisons test). Real-time PCR analysis of Th1-inducing cytokine *IL-12p40* in fibrin-stimulated BMDMs in the presence or absence of anti-CD11b antibody. Data are presented as mean±s.e.m. (*n*=3–4 independent experiments, *P<0.05, **P<0.01 by one-way ANOVA and Bonferroni's multiple comparisons test). (**c**) Real-time PCR analysis of transcription factors and cytokines indicative of Th1, Th2, Th17 and T_reg_ cells in the corpus callosum at 3 days after ACSF or fibrinogen injection. Data are presented as mean±s.e.m. (*n*=3–5 mice per group). *P<0.05 (non-parametric Mann–Whitney *U*-test). (**d**) Comparison of IFN-γ-, IL-4- and IL-17-producing cells in infiltrated CD4^+^ and CD8^+^ T cells isolated from the brains and spleens after ACSF or fibrinogen injection at day 7 post injection. Data are presented as mean±s.e.m. (*n*=3 independent experiments, *P<0.05, ***P<0.001 by one-way ANOVA and Bonferroni's multiple comparisons test). (**e**) IFN-γ and IL-4 expression in CD4^+^ lymph node T cells isolated from saline- or fibrin-depleted (ancrod) mice after PLP_139–151_-induced EAE and restimulated *in vitro* with PLP_139–151_. Data are presented as mean±s.e.m. (*n*=6 mice per group, *P<0.05, **P<0.01 by non-parametric Mann–Whitney *U*-test). ANOVA, analysis of variance; FACS, fluorescence-activated cell sorting; T_reg_, regulatory T cell.

T-cell differentiation into either Th1 or Th17 cells is central to the induction of autoimmune demyelination, and is pertinent to MS pathogenesis^39^,^40^. In a co-culture system of CD4^+^ T cells with BMDMs stimulated with a fibrin preparation, we observed elevated expression of Th1 (*T-bet*), but not Th2 (*Gata-3*) or Th17 (*Rorc*) transcription factors (Fig. 5a). In accordance, the frequency of interferon-γ (IFN-γ)^+^CD4^+^ T cells, but not interleukin (IL)-4^+^CD4^+^ T cells, was higher when co-cultured with fibrin-stimulated BMDMs (Fig. 5b). Treatment of fibrin-stimulated BMDMs with an antibody against CD11b (M1/70) reduced the frequency of IFN-γ^+^ CD4^+^ T cells and the gene expression of *IFN-*γ compared with treatment with isotype control antibody (Fig. 5b). Fibrin stimulation of BMDMs increased expression of the Th1-promoting cytokine *IL-12p40* (ref. 41), which was markedly blocked by anti-CD11b treatment (Fig. 5b). Co-culture of BMDMs stimulated with kininogen did not induce Th1-cell differentiation (Supplementary Fig. 11). Introduction of fibrinogen in the CNS increased expression of the Th1 transcription factor, *T-bet*, but not the Th2, Th17 or regulatory T-cell transcription factors, *Gata-3*, *Rorc* and *FoxP3*, respectively (Fig. 5c). In accordance, fibrinogen injection increased gene expression of the Th1 cytokine *IFN-γ* and *IL-12p40*, while it had no effect on the expression of *IL-4* (Th2) and *IL-17F* (Th17) (Fig. 5c). T cells isolated from the fibrinogen-injected corpus callosum also displayed increased IFN-γ-expressing cells among infiltrated CD4^+^ and CD8^+^ T cells by 6.19- and 28.2-fold, respectively (Fig. 5d). In contrast, fibrinogen injection did not significantly increase the number of IL-4^+^ or IL-17^+^ in CD4^+^ and CD8^+^ T cells as compared with ACSF injection (Fig. 5d). Fibrinogen induced local Th1 polarization specifically in the brain, as no changes were observed in the spleen (Fig. 5d). In accordance, depletion of fibrinogen in EAE reduced the number and proliferation of IFN-γ^+^ T cells, while no effects were observed in IL-4^+^ T cells (Fig. 5e). Overall, these findings introduce the novel concept that a plasma-derived coagulation factor, fibrinogen, may be a key molecular pathway to selectively induce release of Th1-polarizing cytokines in APCs and increase the effector function of encephalitogenic T cells.

### Fibrinogen induces macrophage recruitment into the CNS

**Figure 6:**
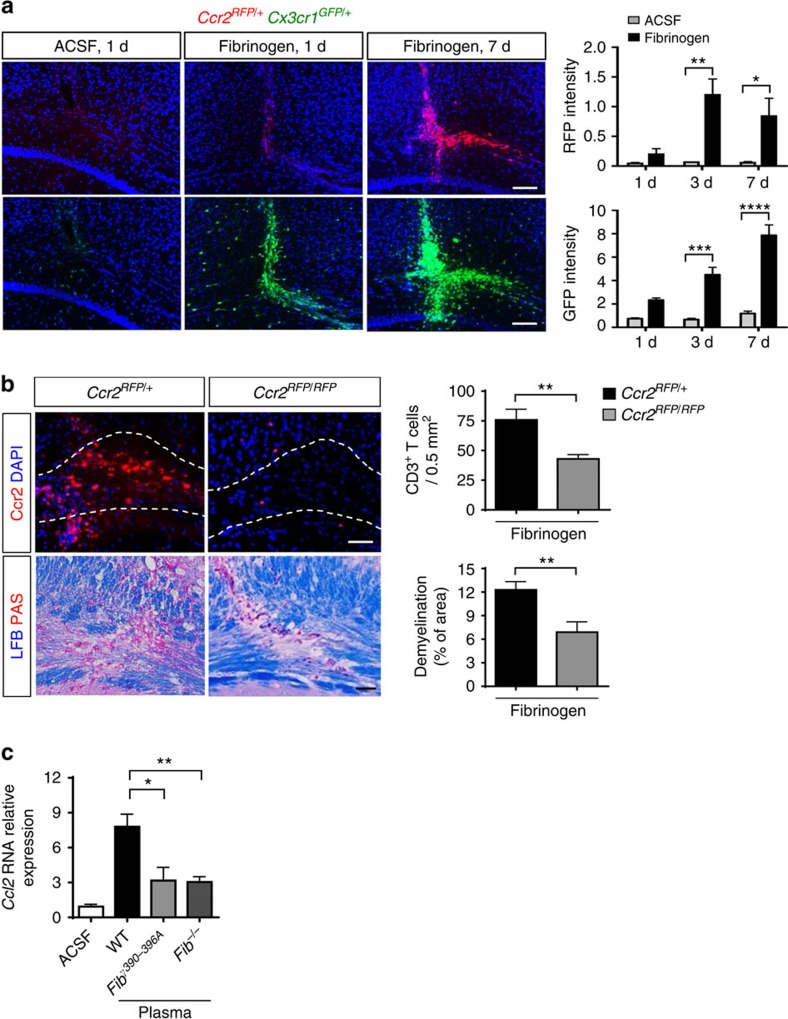
Fibrinogen induces recruitment of peripheral macrophages into the CNS via CD11b/CD18-mediated upregulation of CCL2. (**a**) Infiltration of peripheral Ccr2^+^ macrophages (RFP, red) in the corpus callosum 7 days after fibrinogen injection in *Ccr2*^*RFP/+*^*Cx3cr1*^*GFP/+*^. Scale bar, 200 μm. Quantification of RFP (top graph) and GFP (bottom graph) intensity in the same sections of corpus callosum of ACSF- or fibrinogen-injected *Ccr2*^*RFP/+*^*Cx3cr1*^*GFP/+*^mice on days 1, 3 and 7. Data are presented as mean±s.e.m. (*n*=4–6 mice per time point). *P<0.05, **P<0.01, ***P<0.001, ****P<0.0001 (two-way ANOVA and Bonferroni's multiple comparisons test). (**b**) Infiltration of peripheral Ccr2^+^ macrophages (RFP, red) in the corpus callosum 7 days after fibrinogen injection in *Ccr2*^*RFP/+*^- and Ccr2-deficient (*Ccr2*^*RFP/RFP*^) mice at 7 days post-fibrinogen injection. Scale bar, 50 μm. Quantification of infiltrated CD3^+^ T cells (top graph) and demyelinated area (bottom graph) in the corpus callosum of *Ccr2*^*RFP/+*^- and Ccr2-deficient (*Ccr2*^*RFP/RFP*^) mice 7 days after fibrinogen injection. Data are presented as mean±s.e.m. (*n*=6–7 mice per group). **P<0.01 (non-parametric Mann–Whitney *U*-test). (**c**) Real-time PCR analysis of *Ccl2* gene expression in corpus callosum 12 h after injection of ACSF and plasma obtained from WT, *Fib*γ^*390–396A*^ *Fib*^−/−^ or mice. Data are presented as mean±s.e.m. (*n*=4 mice per group). *P<0.05, **P<0.01 (one-way ANOVA and Bonferroni's multiple comparisons test). ANOVA, analysis of variance; d, days.

Recruitment of CCR2^+^ peripheral monocytes regulate the severity of demyelination^26^,^42^, and are essential for EAE progression to paralytic disease^43^,^44^. We injected fibrinogen into *Ccr2*^*RFP/+*^*Cx3cr1*^*GFP/+*^ mice, which differentially label resident microglia (green fluorescent protein (GFP) positive (GFP^+^), green) and inflammatory monocytes (red fluorescent protein (RFP) positive (RFP^+^), red)^42^. RFP^+^ cells were detected at 3 days after injection and their numbers remained elevated at 7 days after injection (Fig. 6a). GFP^+^ cells rapidly accumulated 1 day after fibrinogen injection and gradually increased up to 7 days post injection (Fig. 6a), suggesting that microglial activation preceded peripheral macrophage cell infiltration. No RFP^+^ cells were found in the corpus callosum after ACSF injection. These results suggest that in addition to T-cell infiltration, extravascular fibrinogen also triggers recruitment of inflammatory monocytes into the CNS.

Fibrinogen increased *Ccl2* in the corpus callosum *in vivo*, and after stimulation of primary microglia and APCs (Fig. 2a–c). Interestingly, the local upregulation of CCL2 appeared to be instrumental in FIE. Indeed, peripheral macrophage recruitment, T-cell infiltration and demyelination were reduced following fibrinogen injection into *Ccr2*^*RFP/RFP*^ mice lacking the CCL2 receptor (Fig. 6b). Like purified fibrinogen, WT plasma was a potent inducer of *Ccl2* and *Cxcl10* expression, whereas injection of either *Fib*^−/−^ or *Fib*γ^*390-396A*^ plasma resulted in their significant reduction (Fig. 6c; Supplementary Fig. 7c). Overall, these results suggest that fibrinogen-induced upregulation of *Ccl2* via CD11b/CD18 facilitates the recruitment of peripheral macrophages and contributes to the induction of inflammatory demyelination.

### CD11b inhibition rescues the fibrinogen CNS effects

**Figure 7:**
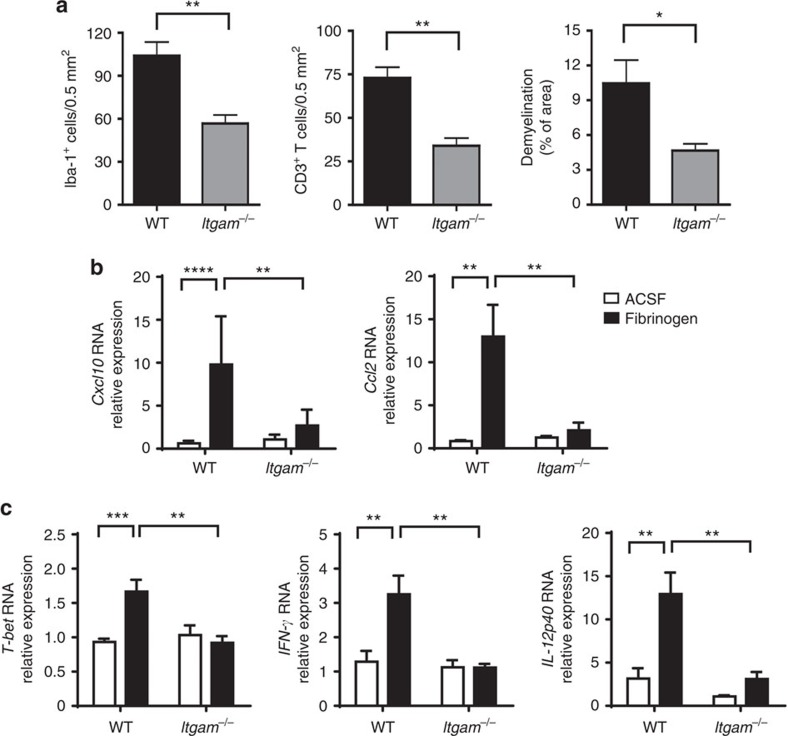
Genetic inhibition of CD11b blocks fibrinogen-induced microglial activation and inflammatory demyelination. (**a**) Quantification of microglial activation (IBA-1), T-cell infiltration (CD3) and demyelination (LFB/PAS) 7 days after fibrinogen injection in the corpus callosum of *Itgam*^−/−^ or control WT mice (*n*=6). Data are presented as mean±s.e.m. *P<0.05, **P<0.01 (non-parametric Mann–Whitney *U*-test). (**b**) Fibrinogen-induced gene expression of *Cxcl10* and *Ccl2* is reduced in the corpus callosum of *Itgam*^−/−^ mice compared with WT control. Results are mean±s.e.m. of 6–7 mice per group, **P<0.01, ****P<0.0001 (two-way ANOVA and Bonferroni's multiple comparisons test). (**c**) Fibrinogen-induced gene expression of *T-bet*, *IFN-*γ and *IL-12p40* is reduced in the corpus callosum of *Itgam*^−/−^ mice compared with WT control (*n*=5–8 mice). Data are presented as mean±s.e.m. **P<0.01, ***P<0.001 (two-way ANOVA and Bonferroni's multiple comparisons test). ANOVA, analysis of variance; d, days; LFB, Luxol fast blue.

**Figure 8:**
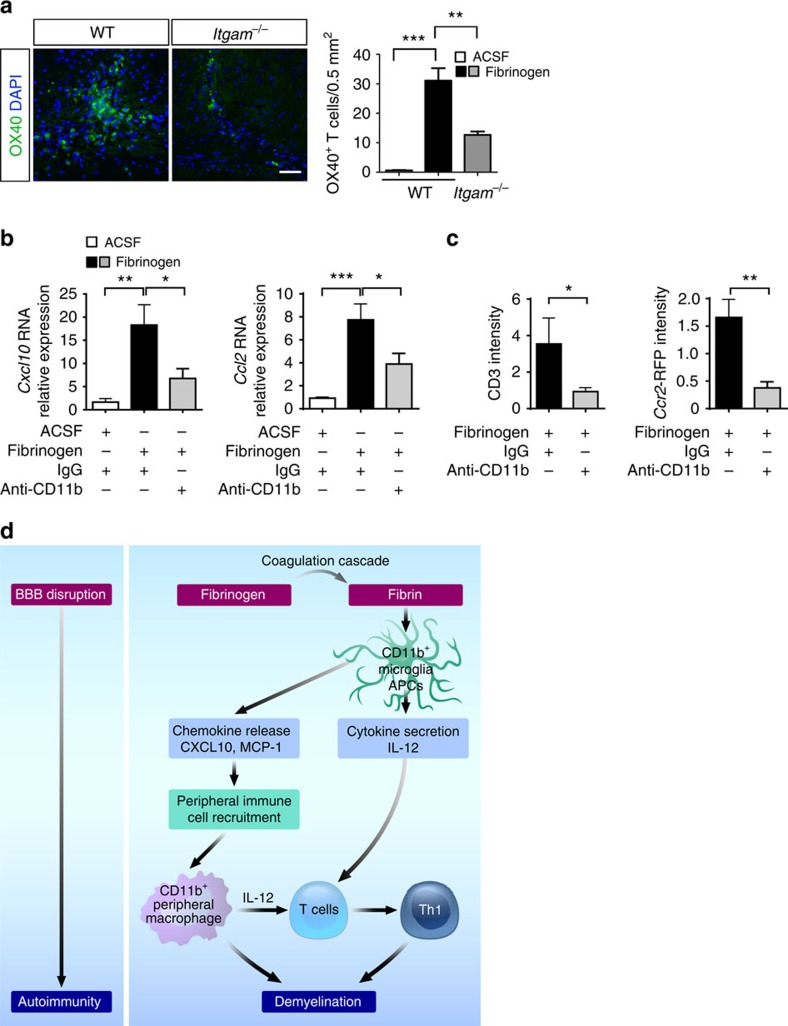
Inhibition of CD11b blocks fibrinogen-induced local T-cell activation, chemokine gene expression and peripheral inflammatory cell recruitment. (**a**) Fibrinogen-induced local T-cell activation (OX-40) in the corpus callosum of WT mice is significantly reduced in *Itgam*^−/−^ mice (*n*=4). Representative images are shown. Data are presented as mean±s.e.m. **P<0.01, ***P<0.001 (one-way ANOVA and Bonferroni's multiple comparisons test). Scale bar, 100 μm. (**b**) *In vivo* pharmacologic blockade of CD11b by intracerebroventricular delivery of anti-CD11b antibody reduces fibrinogen-induced *Cxcl10* and *Ccl2* gene expression, compared with isotype IgG control antibody. Data are presented as mean±s.e.m. (*n*=5 per group). *P<0.05, **P<0.01, ***P<0.001 (one-way ANOVA and Bonferroni's multiple comparisons test). (**c**) Quantification of infiltrated CD3^+^ T cells and RFP^+^ macrophages in the corpus callosum 7 days after injection of fibrinogen in WT mice treated with anti-CD11b or IgG isotype control antibody. Data are presented as mean±s.e.m. (CD3, *n*=6–7 mice per group; RFP, *n*=7 mice per group). *P<0.05, **P<0.01 (non-parametric Mann–Whitney *U*-test). (**d**) Proposed model for the role of fibrin, the final product of the coagulation cascade, in the development of CNS autoimmunity. On BBB disruption, fibrinogen extravagates into the CNS and is converted to fibrin upon activation of coagulation. Fibrin, the high-affinity plasma-derived ligand for CD11b/CD18, activates CNS resident innate immune cells (microglia and perivascular macrophages) to stimulate chemokine release leading to recruitment of peripheral inflammatory macrophages/monocytes and T cells. Fibrin also induces antigen-presenting properties and provides instructive signals (such as IL-12) for inducing Th1-cell differentiation. Fibrin-induced microglial activation, recruitment of peripheral macrophages and T-cell activation lead to inflammatory demyelination. ANOVA, analysis of variance.

To further examine whether CD11b/CD18 signalling is required for fibrinogen-induced demyelination, we injected fibrinogen into the corpus callosum of CD11b-deficient mice (*Itgam*^−/−^)^45^. Microglial activation, T-cell infiltration, demyelination and expression of *Cxcl10*, *Ccl2, T-bet*, *IFN-*γ and *IL-12p40* were significantly decreased in *Itgam*^−/−^ mice after fibrinogen injection, compared with WT (Fig. 7), suggesting the requirement for CD11b/CD18 signalling in fibrinogen-induced adaptive immune activation. Expression of OX-40, a co-stimulatory molecule expressed in activated T cells^46^, increased by ∼30-fold is on CNS-infiltrating T cells after fibrinogen injection (Fig. 8a). OX-40-labelled T-cell activation was significantly reduced in *Itgam*^−/−^ mice after fibrinogen injection by ∼60% (Fig. 8a), suggesting that fibrinogen induces local CNS T-cell activation in a CD11b-dependent manner. Consistent with these results, in mice challenged by fibrinogen injection into the corpus callosum pharmacologic inhibition of CD11b by intracerebroventricular delivery of M1/70 reduced expression of *Cxcl10* and *Ccl2* (Fig. 8b), and decreased T-cell and peripheral macrophage infiltration (Fig. 8c), when compared with isotype control IgG-treated cohorts. Taken together, these data suggest that the introduction of fibrinogen into CNS tissues induces peripheral immune cell recruitment, T-cell activation and demyelination via a CD11b/CD18-dependent mechanism.

## Discussion

This study establishes the fundamental role of the coagulation cascade as a driver of adaptive immune responses and the mechanisms by which extravasation of a blood clotting factor into the brain white matter can induce autoimmunity, demyelination and peripheral macrophage recruitment into the CNS. Our results suggest that fibrin, the final product of the coagulation cascade, constitutes a sustained, non-diffusible and geographically constrained immunological molecular switch that triggers the inopportune local activation of resident APCs to induce the proliferation, recruitment and local activation of myelin antigen-specific Th1 cells in the CNS. The introduction of fibrinogen in myelinated areas in the healthy CNS led to the four major findings (Fig. 8d): (i) fibrinogen leakage in the CNS is a major plasma protein that induces CNS autoimmune responses and demyelination; (ii) fibrinogen is a key inducer of recruitment of both encephalitogenic T cells and peripheral macrophages into the CNS via CXCL10 and CCL2 chemokine secretion, respectively; (iii) activation of the coagulation cascade in the CNS favours Th1-cell differentiation via fibrin-induced upregulation of IL-12; and (iv) the effects of coagulation to CNS autoimmunity are primarily due to the proinflammatory, CD11b-mediated effects of fibrin, thus dissecting the beneficial prohaemostatic roles of coagulation from its damaging effects in CNS disease. Fibrinogen is not only sufficient to induce T-cell activation and demyelination in the healthy CNS, but is also required for T-cell activation after peripheral autoimmune activation as shown on depletion of endogenous fibrinogen in EAE. These new findings, together with our prior studies in EAE showing (i) early activation of coagulation^4^ and (ii) reduction of neurological signs, microglial activation, demyelination and axonal damage on fibrinogen depletion^5^,^11^,^14^, bolster the significance of coagulation activity in the development of neuroinflammatory lesions and establish fibrinogen as a key component of the coagulation cascade with pleiotropic functions in CNS innate and adaptive immunity.

In EAE, T cells enter the CNS after local activation by perivascular macrophages at the leptomeninges and their initial local CNS activation determines the clinical outcome of the autoimmune response^18^. Fibrin is localized in the leptomeninges at EAE pre-onset and induces early perivascular clustering of microglia and meningeal macrophages^5^. Moreover, in marmoset EAE early BBB leakage is associated with perivascular inflammatory cuffing and parenchymal microglial activation, but precedes demyelination^6^. Therefore, it is possible that within the CNS fibrin functions as an instructive signal enabling antigen-presenting properties in resident perivascular macrophages and thus facilitating T-cell entry, proliferation and activation. MOG_35–55_ tetramer binding in the cervical lymph nodes (Fig. 4b) and MOG_35–55_-induced BrdU incorporation (Fig. 4c) demonstrate the presence of functional autoreactive myelin antigen-specific CD4^+^ T cells in C57BL/6 mice injected with fibrinogen in the corpus callosum. Future studies will characterize the responses of T cells to other myelin antigens of not only C57BL/6 mice, but also in the CNS and draining lymph nodes of other mouse strains. Since interactions of T cells with APCs also continue during the peak of EAE^18^, fibrin-induced activation of autoimmune responses might play a role not only at the onset but also for the amplification and perpetuation of the autoimmune response.

Strikingly, our study shows that extravascular fibrinogen induces a transcriptional program of immune effector–cell activation and recruitment that links innate with adaptive immunity. Upregulation of CXCL10 and IL-12p40, two immune modulators involved in T-cell recruitment and Th1-cell differentiation, occurs before the infiltration of peripheral leukocytes, suggesting that fibrinogen-induced activation of CNS innate immunity appears to be a primary pathogenic event that precedes T-cell entry into the CNS. Since CCL2-dependent recruitment of peripheral monocytes is required for antigen-specific Th1 immune responses^26^, fibrin-induced recruitment of peripheral monocytes might contribute to Th1-cell differentiation. Indeed, the fibrin-induced *IL-20p40* expression in both microglia and BMDMs observed here suggests that both resident and peripheral CD11b^+^ innate immune cells can be activated by fibrin to enable autoimmune responses. Therefore, an attractive scenario based on the present findings is that weakening of the BBB either by peripheral brain autoimmune response or by exposure to high-risk environmental factor(s) that weaken the BBB, such as systemic inflammation and infection, extravascular fibrin deposition and activation of coagulation in the CNS might be key upstream signals for the activation of innate and adaptive immune responses.

The time-course study in *Ccr2*^*RFP/+*^*Cx3cr1*^*GFP/+*^ mice showed that the primary effect of fibrinogen is on the resident GFP^+^ microglia and secondarily on the peripheral RFP^+^ macrophages (Fig. 6). Indeed, morphologic changes in GFP-expressing microglia occur as early as 1 day after fibrinogen injection, while RFP-expressing macrophages are not detected in the CNS until 3 days after fibrinogen injection (Fig. 6a). Accordingly, increased chemokine gene expression in the corpus callosum is detected as early as 12 h after fibrinogen injection (Fig. 2a), before the infiltration of peripheral macrophages (Fig. 6a) and T cells (Fig. 1a). Notably, pharmacologic inhibition of CD11b in the CNS by intracerebroventricular delivery of M1/70 reduced chemokine expression and decreased peripheral macrophage infiltration (Fig. 8b,c), further suggesting that microglia are the primary cell target of fibrinogen *in vivo*. CD11b/CD18 is expressed in microglia and macrophages and regulates myelin phagocytosis^11^. It is possible that CD11b/CD18 engagement of extravascular fibrinogen induces activation of APC properties in innate immune cells in a hierarchical manner, first by activating microglia and then peripheral macrophages after they enter the CNS. Since peripheral macrophages enter into the CNS in neurodegenerative diseases associated with BBB and fibrin deposition, such as stroke and Alzheimer's disease^47^, fibrinogen might stimulate the recruitment of peripheral macrophages into the CNS in a wide spectrum of neurological diseases.

We established FIE as a novel experimental setting for exploring the cascade of pathogenic events that directly follow from the leakage of plasma proteins in the white matter in the absence of pre-existing peripheral immune activation or myelin pathology. We selected stereotactic delivery of fibrinogen as it is a common experimental method in viral- and toxin-induced demyelination^48^, as well as in established neurodegeneration models, such as kainic acid injection in the hippocampus. Moreover, stereotactic delivery evades any complexities associated with the imposed genetic expression, assembly and processing of all three chains of fibrinogen. FIE could be used to dissect the contribution of BBB disruption and the coagulation cascade in the CNS without the confounding factors of pre-existing viral, cytokine, toxin or autoimmune events. Given its rapid 7-day disease course, FIE could also be used as a pharmacodynamic model to rapidly test the efficacy of novel treatments for inhibiting CNS-targeted innate and adaptive immune responses. Using albumin, kininogen and plasma derived from *Fib*^−/−^ and *Fib*^*γ377-395*^ mice, we show that fibrinogen is a major protein in the blood that drives sustained neuroinflammatory responses in the CNS. It is possible that in addition to fibrinogen, other plasma proteins might play a role in brain pathology. In particular, other components of the coagulation cascade involved in fibrin formation and clot stabilization, such as thrombin, factors X, XII and XIII could be involved in inflammatory responses^49^. Future studies will elucidate the relative contribution of blood proteins to brain inflammation and neurodegeneration. Our study demonstrates that fibrinogen induces demyelination using myelin-specific antibodies and histological stains that clearly show myelin loss. Demyelination can be associated with axonal damage in MS and Alzheimer's disease^50^,^51^,^52^. Future studies will show whether fibrinogen-induced demyelination is primary associated with preservation of axons, or whether it is accompanied by axonal damage.

Our results may have broad implications for the potential development of new therapeutic strategies for neuroinflammatory diseases. Activation of CD11b^+^ resident CNS cells appear to be one of the earliest events leading to local CNS antigen presentation, amplification of myelin-reactive T-cell responses, infiltration of peripheral macrophages and axonal damage^5^,^17^,^53^,^54^. Since CD11b/CD18 is a pleiotropic receptor^55^, specific inhibition of its pathogenic ligand fibrinogen would be a preferred upstream therapeutic strategy for suppressing the pathogenic cascade in neuroinflammation over any global inhibition of CD11b/CD18. Selective disruption of CD11b/CD18–fibrinogen interface would also be expected to protect the brain from axonal damage and neurodegeneration^5^. Importantly, targeting the interaction of fibrinogen with CD11b would not affect the beneficial functions of fibrinogen in haemostasis^1^,^11^. Since activation of innate immunity is a hallmark of neuroimmune and neurodegenerative diseases, inhibiting the interaction of fibrinogen with CD11b/CD18 could be beneficial not only for suppressing autoimmunity, but also halting neurodegeneration. Identification of extravascular fibrinogen as a key regulator of CNS innate and adaptive immunity might allow us to develop novel therapies targeting early and late events in neuroinflammatory diseases and potentially provide new treatment options.

## Methods

### Mice

C57BL/6 *MHC II*^−/−^ (ref. 28), *Cxcl10*^−/−^ (ref. 29), *Itgam*^−/−^ (ref. 45)*, Cx3cr1*^*GFP/+*^ (ref. 56), MOG-specific TCR transgenic mice (2D2)^31^ and SJL/J were purchased from the Jackson Laboratory. C57BL/6 *Fib*^−/−^ (ref. 19), *Fib*^*γ390–396Α*^ mice^22^, OVA-specific TCR-transgenic mice (OT-II)^32^, *RAG1*^−/−^ (ref. 57), *RAG2*^−/−^*γc*^−/−^ (ref. 30) and *Ccr2*^*RFP/RFP*^ mice^42^ were also used. C57BL/6 *Ccr2*^*RFP/RFP*^ mice were crossed with *Cx3cr1*^*GFP/GFP*^ mice to generate *Cx3cr1*^*GFP/+*^*Ccr2*^*RFP/+*^ mice. All animal experiments were performed on adult male mice at 10–15 weeks of age. Mice were housed in the groups of five per cage under standard vivarium conditions and a 12-h light/dark cycle. All animal protocols were approved by the Committee of Animal Research at the University of California, San Francisco, and in accord with the National Institutes of Health guidelines.

### Stereotactic injections in the corpus callosum

Mice were anaesthetized with avertin and placed in a stereotactic apparatus. Plasma plasminogen-free fibrinogen (Calbiochem) was dissolved in endotoxin-free distilled water (HyClone), diluted to 5 mg ml^−1^ with ACSF. Fibrinogen (1 μl of 5 mg ml^−1^), ACSF, albumin (1 μl of 5 mg ml^−1^) or kininogen (1 μl of 0.1 mg ml^−1^) were slowly injected (0.3 μl min^−1^) with a 10-μl Hamilton syringe attached to a 33-G needle into the brain at coordinates (anteroposterior, −1.0 mm; mediolateral, −1.0 mm; dorsoventral, −1.75 mm from the bregma, according to Paxinos and Watson) as described^5^. Plasma was isolated and injected as described^5^.

### Intracerebroventricular delivery of antibody

Functional-grade purified anti-CD11b (M1/70; eBioscience), or isotype control IgG (eBioscience), was injected (0.2 μl min^−1^) with a 10-μl syringe attached to a 33-G needle into cerebral ventricle (anteroposterior, −2.0 mm; mediolateral, 0 mm, dorsoventral, −2.0 mm) 30 min before fibrinogen injection.

### Stereotactic injections in the spinal cord

Mice were anaesthetized by intraperitoneal injection of ketamine (100 mg kg^−1^) and xylazine (15 mg kg^−1^). A midline skin incision was made over the upper lumbar regions of spinal cord. The spinal column was secured via the mouse vertebral clamps fixed in a stereotaxic frame. The epidural space was exposed by disruption of the L1–L2 interspinous ligament without laminectomy as described^58^. A pulled glass micropipette prefilled with fibrinogen was inserted into the spinal cord at coordinates (0.3-mm lateral to the spinal midline, a depth of 0.9 mm from the spinal cord surface). Fibrinogen (1 μl of 5 mg ml^−1^) was injected (0.2 μl min^−1^) and the glass micropipette remained in place for 5 min before slowly withdrawn. After surgery, the muscles and skin were sutured, and mice were allowed to recover.

### Expression of recombinant fibrinogen

For the production of recombinant fibrinogen, freestyle HEK293T cells were transiently co-transfected with three eukaryotic expression vectors containing full-length complementary DNA (cDNA) for the three chains of fibrinogen, alpha (1.9 kb), beta (1.3 kb) and gamma (1.3 kb), according to the standard procedures. At day 4 post transfection, medium was collected and the supernatant was passed through a Vivaspin-20 concentrator with cutoff 100,000 molecular weight cutoff and was added to a Slide-A-Lyzer Dialysis cassette with cutoff 10,000 molecular weight cutoff for overnight dialysis in PBS buffer at 40 °C with stirring. Expression of the recombinant fibrinogen protein was evaluated by SDS–polyacrylamide gel electrophoresis gel electrophoresis and Coomassie staining.

### Histology and immunohistochemistry

Mice were transcardially perfused with 4% paraformaldehyde under avertin anaesthesia. Brains and spinal cord were removed, postfixed, processes for paraffin embedding. Luxol fast blue staining and immunohistochemistry were performed as described^11^,^59^,^60^. For toluidine blue staining, animals were intracardially perfused under deep ether anaesthesia with ice-cold 2% paraformaldehyde, 0.5% glutaraldehyde in 0.1 mol l^−1^ phosphate buffer, pH 7.2, for 1 min, followed by ice-cold 3% glutaraldehyde in 0.1 mol l^−1^ phosphate buffer for 5 min. Brains were removed, immersion-fixed for 24 h in phosphate-buffered 3% glutaraldehyde, postfixed in 2% osmium tetroxide solution and subsequently embedded in epoxy resin. Semi-thin sections were cut and stained with toluidine blue. For immunohistochemistry, sections were permeabilized in 0.1% Triton X-100, blocked with 5% bovine serum albumin and 5% normal donkey serum, and incubated for 24 h at 4 °C with primary antibodies. Primary antibodies were rabbit anti-Iba-1 (1:1,000, Wako), rabbit anti-CD3 (1:1,000, Dako), rabbit anti-GFAP (1:500, Sigma), mouse anti-MBP (1:100, Covance), rat anti-mouse MHC class II (1:200, BMA Biomedicals) or goat anti-mouse CXCL10 (1:200, R&D system). Sections were rinsed in PBS with 0.1% Triton X-100 and incubated with secondary antibodies conjugated with Alexa Fluor 488 or 594 (1:200, Jackson Immunochemicals) for 1 h in the dark. Immunohistochemistry using the primary anti-MBP monoclonal antibody was performed with the MOM kit (Vector) according to the manufacturer's instruction. After washing in PBS, sections were mounted on glass slides and coverslipped with Prolong Gold antifading agent (Invitrogen). Images were acquired using an Axioplan II epifluorescence microscope (Zeiss) equipped with dry Plan-Neofluar objectives (10 × 0.3 numerical aperture (NA), 20 × 0.5 NA or 40 × 0.75 NA). Quantification was performed on thresholded images using ImageJ by blinded observers.

### Flow cytometry

Mice were perfused with saline and brain slices were prepared using the adult mouse brain slicer matrix (Zivic Instruments). Corpus callosal area was dissected from the slices and minced tissue was digested using collagenase IV (Roche) at 37 °C for 30 min. Cell suspensions were filtered through a 70-μm-cell strainer (BD Falcon). Cell suspensions were prepared in RPMI-1640 (Invitrogen), supplemented with 5% (vol/vol) heat-inactivated fetal bovine serum (FBS, Invitrogen), 50 U penicillin–streptomycin (Invitrogen) and 50 μM β-mercaptoethanol (Invitrogen). Single-cell suspensions were incubated with Myelin Removal Beads (Miltenyi Biotec), and cells were collected by autoMACs Pro Separator (Miltenyi Biotec). Intracellular staining of splenocytes derived from mice undergoing MOG_35–55_ EAE^5^,^11^ was used as positive control for fluorescence-activated cell sorting antibody staining. For cytokine analysis, cells were incubated for 4 h with phorbol 12-myristate 13-acetate (50 ng ml^−1^, Sigma), ionomycin (500 ng ml^−1^, Sigma) and Golgi-Plug (BD Biosciences) and surface stained with anti-CD3-PE (eBioscience), anti-CD4-APC-Cy7 (eBioscience) and anti-CD8-PacBlue (eBioscience). Cells were then fixed with Cytofix/Cytoperm solution (BD Biosciences), and intracellular cytokine staining was performed with anti-IFN-γ-PE-Cy7 (eBioscience), anti-IL-4-Alexa 647 (eBioscience) and anti-IL-17a-FITC (eBioscience). All labelled antibodies were used at 1:300 dilutions. Flow cytometric analysis was performed on an LSR II (BD Biosciences). Data were analysed using the FlowJo software (Tree Star).

### Detection of endogenous MOG antigen-specific T cells

Fibrinogen-injected brains and its draining lymph nodes were removed at 7 days post injection, and single-cell suspensions were prepared as described above. Prepared lymphocytes were stimulated with 20 μg ml^−1^ MOG_35–55_ for 7 days. Endogeneous MOG antigen-specific T cells were identified with MOG_35–55_-specific T-select I-A^b^ MOG_35–55_ tetramer (MBL). I-A^b^ OVA_323–339_ Tetramer (MBL) was used as a negative control. Cultured cells were resuspended in FCM buffer (2% FCS/0.05% NaN_3_/PBS) and incubated with FcR-blocking antibody for 5 min at room temperature. Cells were incubated with I-A^b^ MOG_35–55_ tetramer for 60 min at 4 °C followed by anti-CD4-FITC incubation for 30 min at 4 °C. After two washes with FCM buffer, cells were analysed with an LSR II.

### T-cell CFSE staining and adoptive transfer

Naive 2D2 CD4^+^ T cells were isolated from spleens and lymph nodes of 2D2 mice using CD4^+^CD62L^+^ T-cell isolation kits (Miltenyi Biotec). Isolated T cells were labelled with CFSE^35^ (Invitrogen) at room temperature. A total of 1 × 10^7^ cells were transferred intraperitoneally into recipient 24 h before ACSF and fibrinogen injection into the corpus callosum. On day 6, labelled T cells were isolated from the brains, and the frequency of CFSE^+^(CFSE_low_)TCR-Vβ11^+^CD4^+^ T cells was analysed by flow cytometry. For analysis of CNS migratory activity of 2D2 or OT-II CD4^+^ T cells, isolated naive 2D2 or OT-II CD4^+^ T cells were co-cultured 3 days with BMDMs incubated with either 20 μg ml^−1^ MOG_35–55_ or OVA_323–339_ peptide together with or without fibrin. Cells were then labelled with CFDA-SE according to the manufacturer's instructions (Invitrogen) and injected (1 × 10^7^ cells per mouse) into the *Rag1*^−/−^ recipient mice that had been injected with fibrinogen 2 days before. Three days after post-T-cell injection, mice were killed, and their brains were subjected to histological analysis.

### Isolation of primary microglia

Microglia were prepared from neonatal rat or mouse pups at postnatal day (P) 2–3. The cortices were separated from meninges and minced with a sterile razor blade. Tissue pieces were transferred into 2.5% Trypsin solution (Life Technologies/Gibco) containing DNAse (SIGMA). After incubation at 37 °C for 25 min the trypsin solution was removed, cortices were washed with 30% FBS in DPBS and serially triturated with 30% FBS in Dulbecco's phosphate-buffered saline (DPBS) (Life Technologies/Gibco) containing DNAse. The cell suspension was gently spun at 200*g* for 15 min and the pellet resuspended in DMEM (Life Technologies/Gibco), containing 10% FBS (Life Technologies/Gibco), 100 units per millilitre penicillin and 100 mg ml^−1^ streptomycin (Life Technologies/Gibco). Cells were plated into poly-D-lysine pre-coated T-75 flasks at a density of 2–3 cortices per flask. On day 3 *in vitro*, fresh medium was added and cells were grown for 1 more day. On day 4 *in vitro*, flasks were placed onto a shaker platform, preheated to 37 °C and microglia cells were shaken off the cortical cell layer at 200 r.p.m. for 2 h. The medium containing mostly microglia cells was removed from the flasks and cells were spun at 200*g* for 15 min. The cell pellets were gently resuspended in culture medium and the microglia density was adjusted to 5000 cells per microlitre.

### Isolation of BMDMs

BMDMs were prepared as described^61^. In brief, bone marrow cells were isolated from tibia and femur of 10-week-old mice and cultured in RPMI-1640 (Invitrogen) supplemented with 10% (vol/vol) heat-inactivated FBS (Invitrogen), 50 U penicillin–streptomycin (Invitrogen), 50 μM β-mercaptoethanol and murine M-CSF (10 ng ml^−1^). On day 6, adherent BMDMs were harvested from plates by the addition of PBS containing 5 mM EDTA for experiments.

### Fibrin stimulation of microglia or BMDMs

To prepare soluble fibrin^62^, human plasma fibrinogen (Calbiochem) was converted to fibrin by mixing with thrombin. Fibrin clots were cut into small pieces and fragmented by sonication. Clot fragments are filtered through sieves, their concentration was determined, and they were stored at −80 °C. Soluble fibrin preparations were tested for LPS and thrombin activity and were shown to have undetectable amounts of contaminants. Cultures were treated with soluble fibrin by adding soluble fibrin to the supernatant. To prepare fibrin-coated plates, a mixture of thrombin (1 U ml^−1^, Sigma) and CaCl_2_ (7 mM, Sigma) in HEPES buffer was added into each well of tissue culture plates (TPP Techno Plastic Products AG, Switzerland) and subsequently 50 μg ml^−1^ of human plasma fibrinogen (Calbiochem) was added. The plates were incubated for 1 h at 37° C. After incubation, solution was evaporated, and moisture retention was minimized by air flow through the dryer system. After washing the plates with PBS, microglia or BMDMs were seeded on fibrin-coated plates. All reagents were made in endotoxin-free water.

### T-cell proliferation assay

CD4^+^ T cells were purified from the spleen with CD4^+^CD62L^+^ T-cell isolation kits (Miltenyi Biotec). CD4^+^ T cells were mixed with BMDM at the ratio (1:5). T-cell proliferation was assessed by BrdU incorporation kit (BD Bioscience) during last 24 h of culture. For antigen-specific T-cell proliferation, CD4^+^ T cells isolated from 2D2 mice were cultured with MOG_35–55_ peptide (20 μg ml^−1^) for 4 days in the presence of BMDM treated with fibrin or primary mouse microglia plated on fibrin-coated plates or stimulated with LPS (200 ng ml^−1^) for 24 h. Cells were then fixed, permeabilized and stained with FITC-conjugated anti-BrdU (BrdU Flow Kits). For measurement of PLP antigen-specific CD4^+^ T-cell proliferation, lymphocytes were isolated from the draining lymph nodes of SJL/J mice at 7 days after fibrinogen and ACSF injection into the corpus callosum. Lymphocytes were stimulated with PLP_139–151_ peptide (20 μg ml^−1^) and PLP_178−191_ peptide (20 μg ml^−1^), and IL-2 (10 ng ml^−1^) was added every 2 days. BrdU incorporation was assessed as described above. For intracellular cytokine staining, CD4^+^ T cells were stimulated with 20 μg ml^−1^ MOG_35–55_ peptide for 72 h and restimulated with PMA and ionomycin for 4 h in the presence of Golgi-Plug (BD Bioscience), after which IFN-γ-, IL-4- and IL-17-producing cells were analysed by intracellular staining.

### RNA isolation and quantitative PCR

RNA was isolated from brain samples with the RNAeasy kit (Qiagen), according to the manufacturer's instructions. RNA was reverse-transcribed to cDNA using the GeneAmp RNA PCR Core kit (Applied Biosystems) and random hexamer primers. Real-time PCR analysis was performed using the Step One Plus (Applied Biosystems) and the Quantitect SYBR Green PCR kit (Qiagen) with 2 μl of cDNA template in a 25-μl reaction. Results were analysed using the Opticon 2 Software and the comparative CT method. Data are expressed as 2^ÄÄCT^ for the experimental gene of interest normalized to the housekeeping gene and presented as fold change relative to control. The gene-specific primers are listed in Supplementary Table 3.

### Gene expression profiling by microarray analysis

Microarray analysis was performed on cultured rat microglia treated with fibrin or BMDMs plated on fibrin-coated plates and brain tissues from ACSF- and fibrinogen-injected mice. Rat pups were used to isolate highly pure microglial cultures in sufficient numbers for microarray analysis. For brain tissue microarray analysis, corpus callosal area was dissected from brain slices prepared with brain slicer matrix (Zivic instruments). Total RNA was isolated using RNeasy Mini kit/RNeasy Lipid tissue mini kit (QIAGEN) according to the manufacturer's instruction. Probes were prepared using NuGEN Ovation Pico WTA V2 kit and NuGEN Encore Biotin Module, and hybridized to Rat and Mouse Gene 1.0 ST GeneChip arrays (Affymetrix). Arrays were scanned using an Affymetrix GCS3000 scanner and Affymetrix Command Console software, and data were normalized using the RMA algorithm in Affymetrix Expression Console. Microarrays were normalized for array-specific effects using Affymetrix's ‘Robust Multi-Array' normalization. Normalized array values were reported on a log2 scale. For statistical analyses, we removed all array probe sets where no experimental groups had an average log2 intensity >3.0. This is a standard cutoff, below which expression is indistinguishable from background noise. Linear models were fitted for each gene using the Bioconductor ‘limma' package in R^63^. Moderated *t*-statistics, fold change and the associated *P* values were calculated for each gene. To account for the fact that thousands of genes were tested, we reported false discovery rate (FDR)-adjusted values, calculated using the Benjamini–Hochberg method^64^. FDR values indicate the expected fraction of falsely declared, differentially expressed (DE) genes among the total set of declared DE genes (that is, FDR=0.15 would indicate that ∼15% of the declared DE genes were expected to be due to experimental noise instead of actual differential expression). The microarray data have been deposited in the Gene Expression Omnibus (GEO) database accession number GSE71084.

### Induction of EAE and systemic fibrinogen depletion

EAE was induced in 8-week-old female SJL/J by subcutaneous immunization with 100 μg PLP_139–151_ (HSLGKWLGHPDKF; Auspep Pty Ltd) as described^11^. Mice were depleted of fibrinogen with ancrod as described^11^. The mice received 2.4 U ancrod per day by mini-osmotic pump. In control animals, buffer-filled minipumps were implanted. On day 9 of immunization, draining lymph node T cells were obtained from EAE mice and cultured with PLP_139–151_ peptide for the detection of intracellular IFN-γ and IL-4 in CD4^+^ T cells and BrdU proliferation assay as described above.

### Statistical analyses

The data are presented as mean±s.e.m. Statistical calculations were performed using the GraphsPad Prism. Data distribution was assumed to be normal, but this was not formally tested. No statistical methods were used to predetermine sample size, but our sample sizes are similar to those reported previously^5^,^11^,^59^. Statistical significance was determined with non-parametric two-sided Mann–Whitney *U*-test, one-way, or two-way, analysis of variance followed by Bonferroni post test (multiple comparisons). Mice and cells were divided into experimental groups in an unbiased manner. No randomization was used to assign groups or collect data. All animals survived until the end of the study and all data points were included in analysis. All histopathological analysis was performed by a blinded observer.

## Additional information

**How to cite this article:** Ryu, J. K. *et al.* Blood coagulation protein fibrinogen promotes autoimmunity and demyelination via chemokine release and antigen presentation. *Nat. Commun.* 6:8164 doi: 10.1038/ncomms9164 (2015).

## Supplementary Material

Supplementary Figures and Supplementary Tables Supplementary Figures 1 -11 and Supplementary Tables 1-3
