# Supplementary material for: Blood coagulation protein fibrinogen promotes autoimmunity and demyelination via chemokine release and antigen presentation
Source: Nat Commun. 2015 Sep 10;6:8164. doi: 10.1038/ncomms9164 (PMC4579523; doi:10.1038/ncomms9164)
Supplement: Supplementary Figures and Supplementary Tables — Supplementary Figures 1 -11 and Supplementary Tables 1-3 [file ncomms9164-s1.pdf]

Supplementary Figure 1

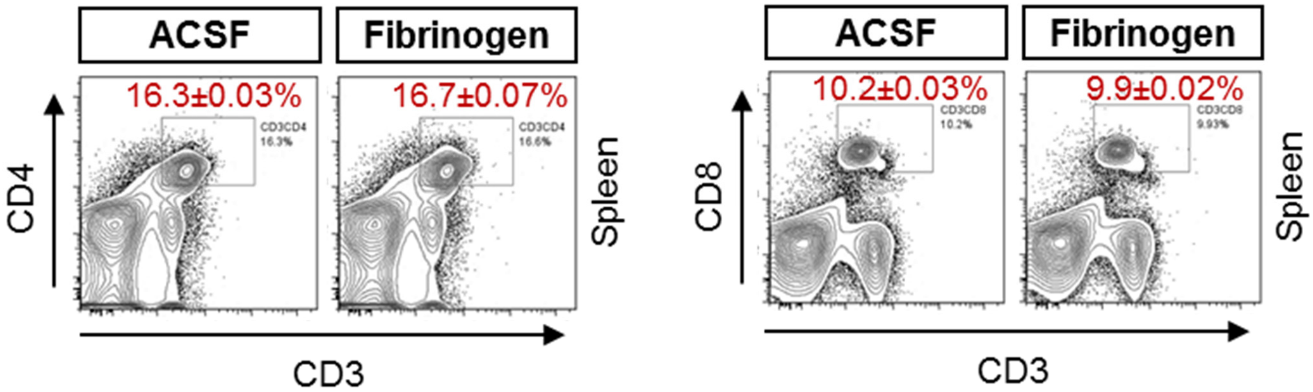

Supplementary Figure 1. Characterization of T cells from the spleen of mice after fibrinogen injection into corpus callosum. FACS analysis of T cells isolated from spleen 7 days after fibrinogen- or ACSF-injection stained with CD3, CD4, and CD8 (*n* = 3 independent experiments; each experiment generated from pooled brain cells from n = 3-4 mice).

Supplementary Figure 2

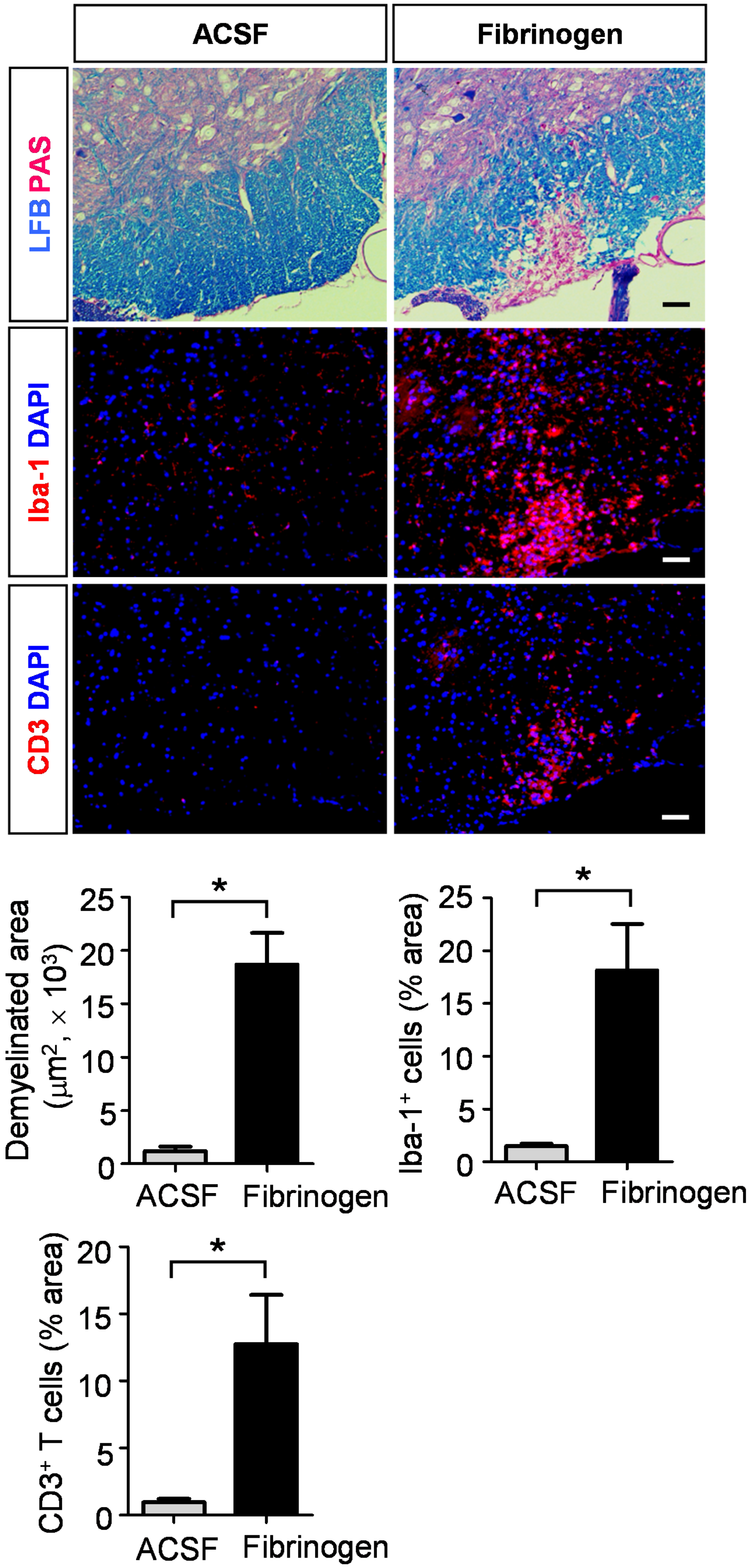

Supplementary Figure 2. Induction of inflammatory demyelination and T cell recruitment after fibrinogen injection into the spinal cord. Demyelination (LFB/PAS, and toluidine blue), microglial activation (Iba-1), and T-cell infiltration (CD3) in the spinal cord of mice 7 days after injection with fibrinogen or ACSF control. Scale bars: 50 μm. Quantification of demyelination, microglial activation, infiltrated T cells in ventral column of spinal cord sections 7 days after ACSF- or fibrinogen-injection. Data are presented as mean ± s.e.m. (n=3–4 mice per group). *$P < 0.05$ (non-parametric Mann-Whitney *U* test).

Supplementary Figure 3

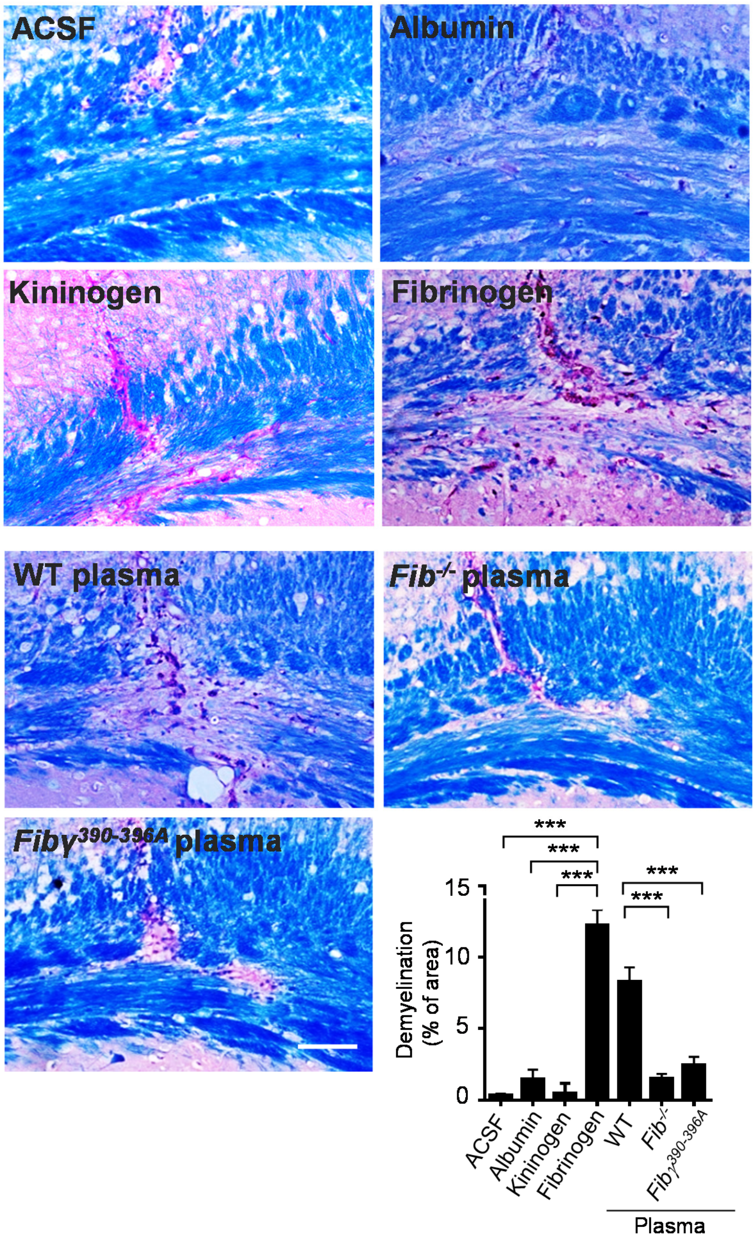

Supplementary Figure 3. Fibrinogen is a major protein in the plasma that induces inflammatory demyelination. Demyelination (LFB/PAS) in the corpus callosum of mice injected with fibrinogen and WT plasma, compared to ACSF, albumin, kininogen or plasma obtained from *Fib*$^{-/-}$ or *Fibγ*$^{390\text{-}396A}$ mice. Scale bar: 100 μm. Quantification of demyelination is shown at 7 days post-injection. Data are presented as mean ± s.e.m. (n = 6 mice per group). ***P < 0.001 (one-way ANOVA).

Supplementary Figure 4

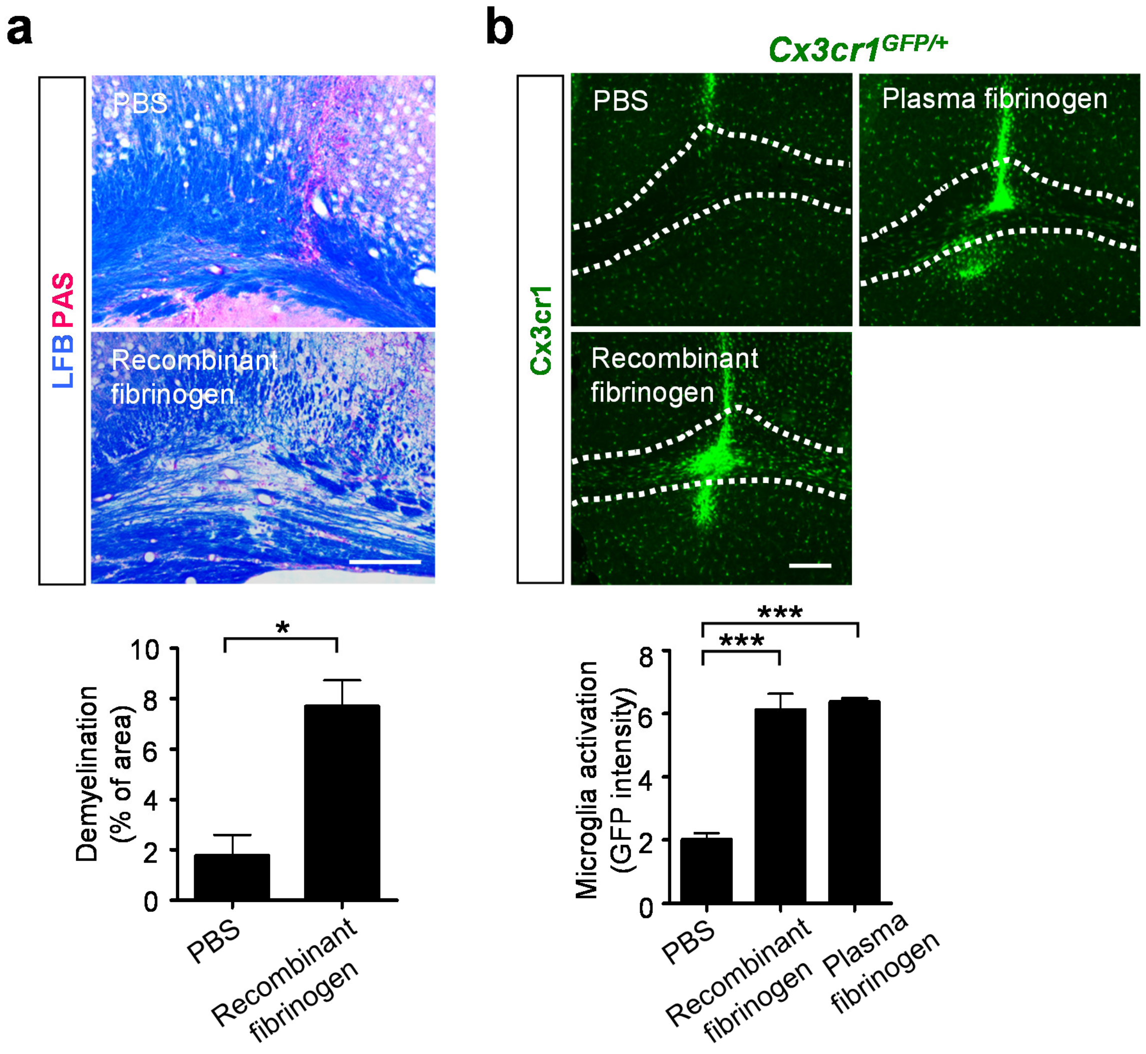

Supplementary Figure 4. Induction of inflammatory demyelination and microglial activation by recombinant fibrinogen injection into the CNS (**a**) Demyelination (LFB/PAS) in the corpus callosum of mice injected with recombinant fibrinogen or PBS control. Scale bar: 300 μm. Quantification of demyelination is shown at 7 days post-injection. Data are presented as mean ± s.e.m. (n=4-5 mice per group). **P* < 0.05 (non-parametric Mann-Whitney *U* test). (**b**) Microglial activation in the corpus callosum of $Cx3CR1^{GFP/+}$ reporter mice after injection of plasma-derived fibrinogen, recombinant fibrinogen, or PBS control. Scale bar: 200 μm. Quantification of microglial activation (GFP intensity) 7 days after injection in the corpus callosum. Data represent mean ± SEM of 4-5 mice per group. ****P* < 0.001 (one-way ANOVA).

Supplementary Figure 5

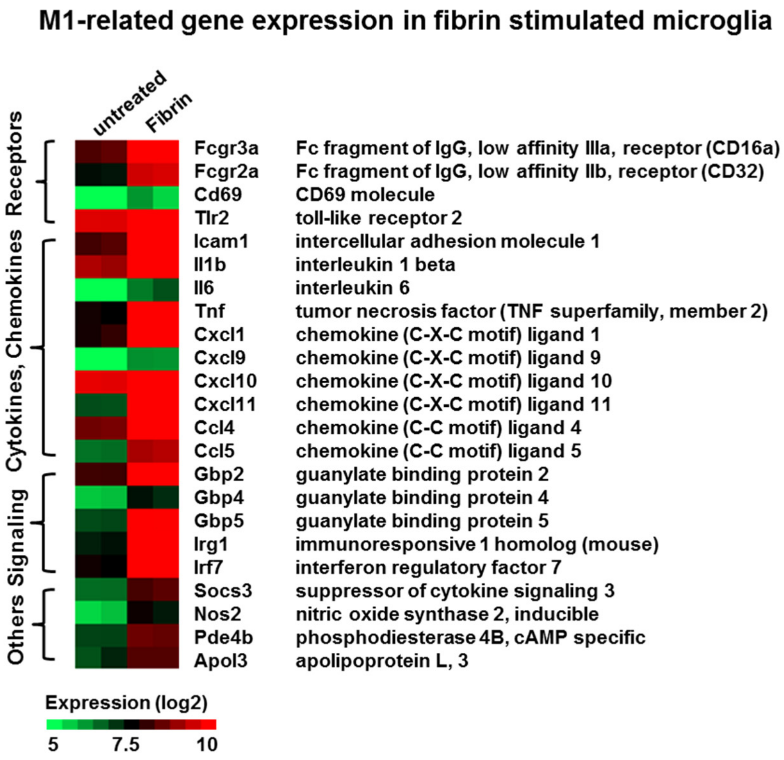

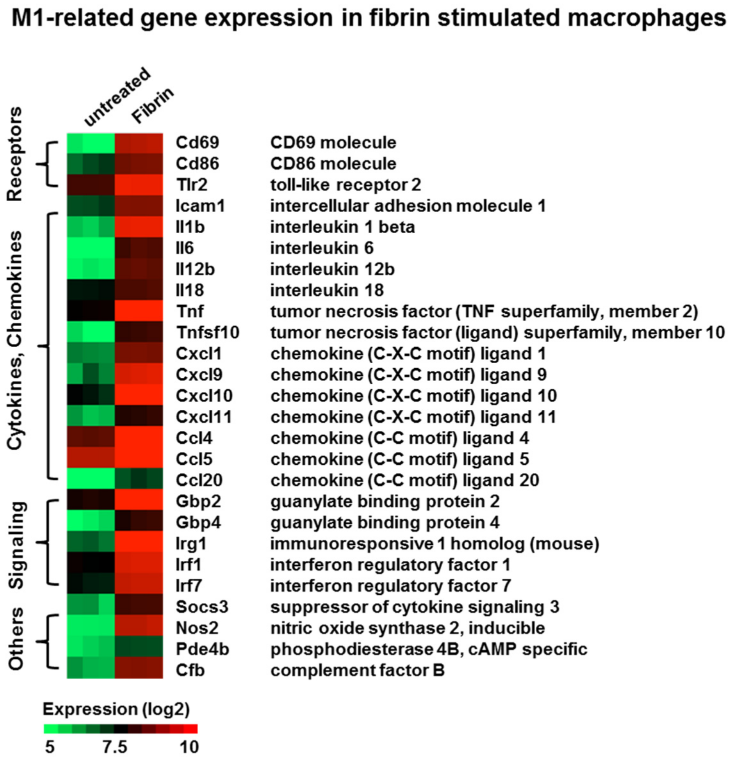

Supplementary Figure 5. Heat map of gene expression for M1-related genes in fibrin-stimulated microglia and macrophages. Affymetrix global gene–expression analysis of fibrin-stimulated microglia (top) and macrophages (bottom) identified M1-related genes differentially expressed between unstimulated and fibrin. The color range indicates the log_2 scale.

Supplementary Figure 6

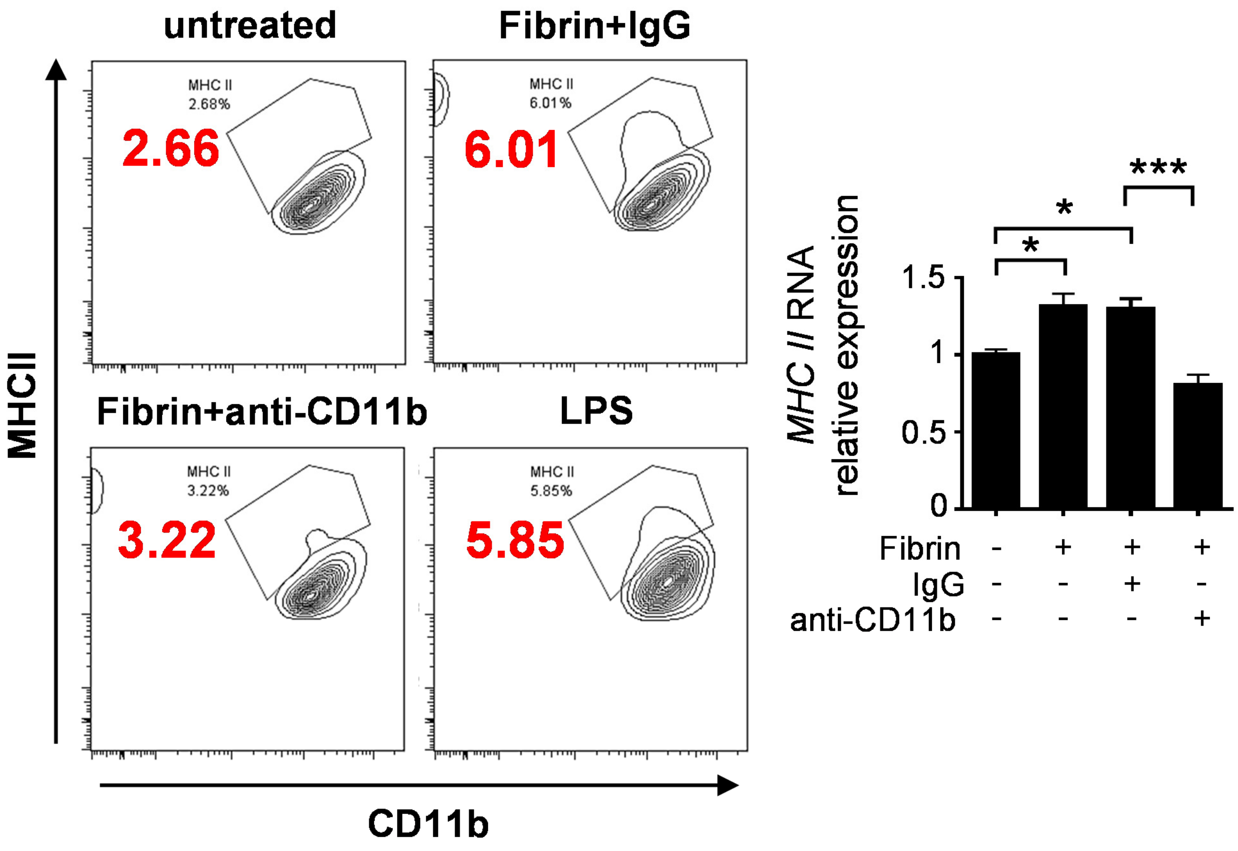

Supplementary Figure 6. Fibrin-induced MHCII expression in APCs. FACS analysis of MHC class II expression in APCs after fibrin stimulation. LPS was used as positive control. Anti-CD11b antibody treatment reduces MHC II$^+$ APCs. Data are presented as mean ± s.e.m. (*n* = 3 independent experiments) (left). Real-time PCR analysis of *MHC class II* gene expression in BMDMs after fibrin stimulation treated with anti-CD11b or IgG isotype control antibody. Data are presented as mean ± s.e.m. (*n* = 4-5 independent experiments) (right). $*P < 0.05$, $***P < 0.001$ (one-way ANOVA and Bonferroni's multiple comparisons test).

Supplementary Figure 7

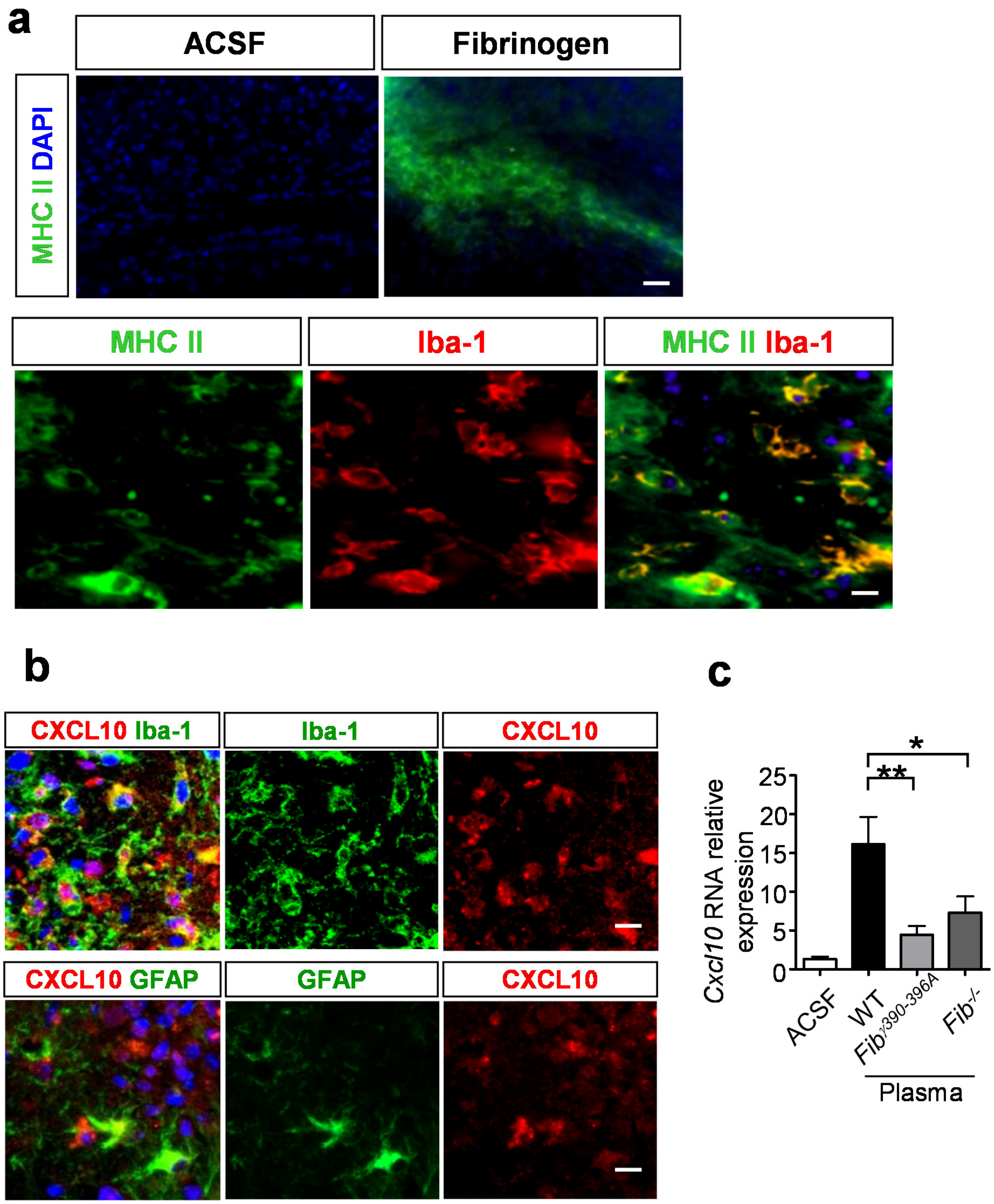

Supplementary Figure 7. Fibrinogen induces expression of antigen presenting molecule MHC class II and chemokine Cxcl10 in microglia in the CNS. (**a**) MHC class II upregulation in the corpus callosum 7 days after injection of fibrinogen compared to ACSF (green, top panels). Double immunofluorescence staining for MHC class II and Iba-1 3 days after fibrinogen injection shows colocalization of activated Iba-1$^+$ microglia (red) with MHC class II (green) in the fibrinogen-injected corpus callosum (yellow, bottom panels). Scale bars: 50 µm (top panel), 40 µm (bottom panel). (**b**) Double immunofluorescence for CXCL10 (red) with the microglial marker Iba-1 (green) or the astrocyte marker GFAP (green) three days after injection of fibrinogen in the corpus callosum. CXCL10 was detected in Iba-1$^+$ microglia (upper panel, yellow) and in few GFAP$^+$ astrocytes (lower panel). Scale bars: 10 µm. (**c**) Real-time PCR analysis of *Cxcl10* gene expression in corpus callosum 12 h after injection of ACSF and plasma obtained from WT, $Fib\gamma^{390\text{-}396A}$ or $Fib^{-/-}$ mice. Data are presented as mean ± s.e.m. (n = 4 per group). *$P < 0.05$, **$P < 0.01$ (one-way ANOVA).

Supplementary Figure 8

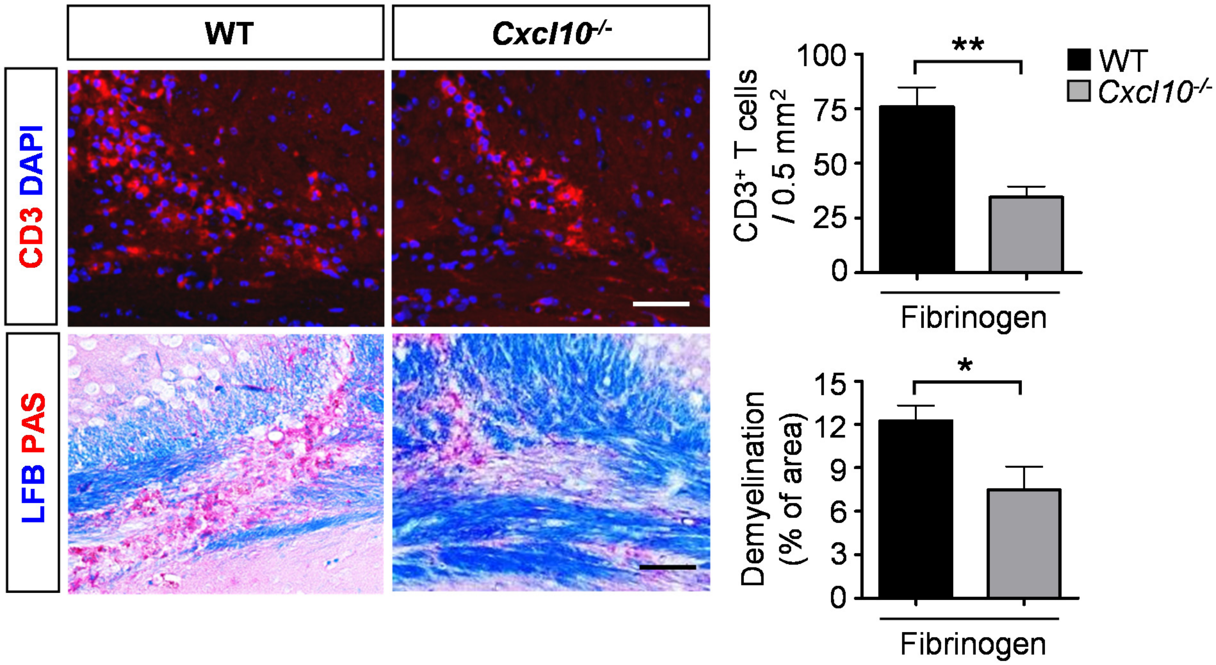

Supplementary Figure 8. Fibrinogen induces T-cell recruitment into the CNS via CD11b/CD18-mediated upregulation of CXCL10. T cell infiltration (CD3) and demyelination (LFB/PAS) in the corpus callosum of WT and *Cxcl10*$^{-/-}$ mice 7 days after injection with fibrinogen. Representative images are shown. Scale bars: 100 μm. Quantification of infiltrated $CD3^{+}$ T cells and demyelinated areas after fibrinogen injection. Data are presented as mean ± s.e.m. ($n = 6$ per group). *$P < 0.05$, **$P < 0.01$ (non-parametric Mann-Whitney *U* test).

Supplementary Figure 9

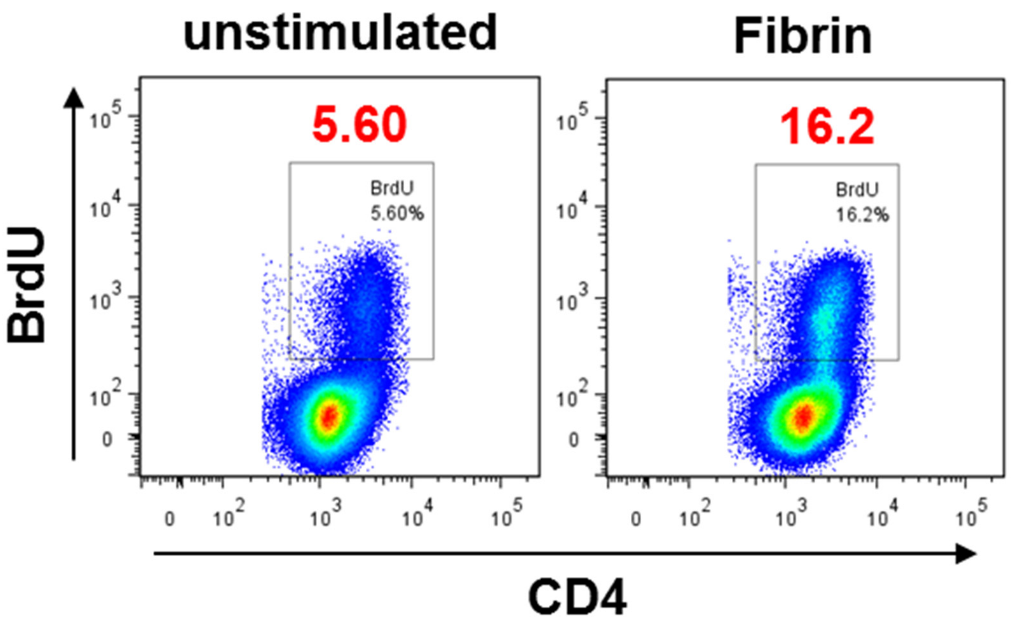

Supplementary Figure 9. Fibrin stimulated microglia increases antigen-specific T cell proliferation. Primary mouse microglia isolated from postnatal brains were treated with fibrin and co-cultured for 4 d with naive $CD4^+$ 2D2 T cells in the presence of 20 μg/ml MOG_{35-55}. Proliferation of T cells was measured by BrdU incorporation during the last 24 h of culture. Data are representative of two independent experiments.

Supplementary Figure 10

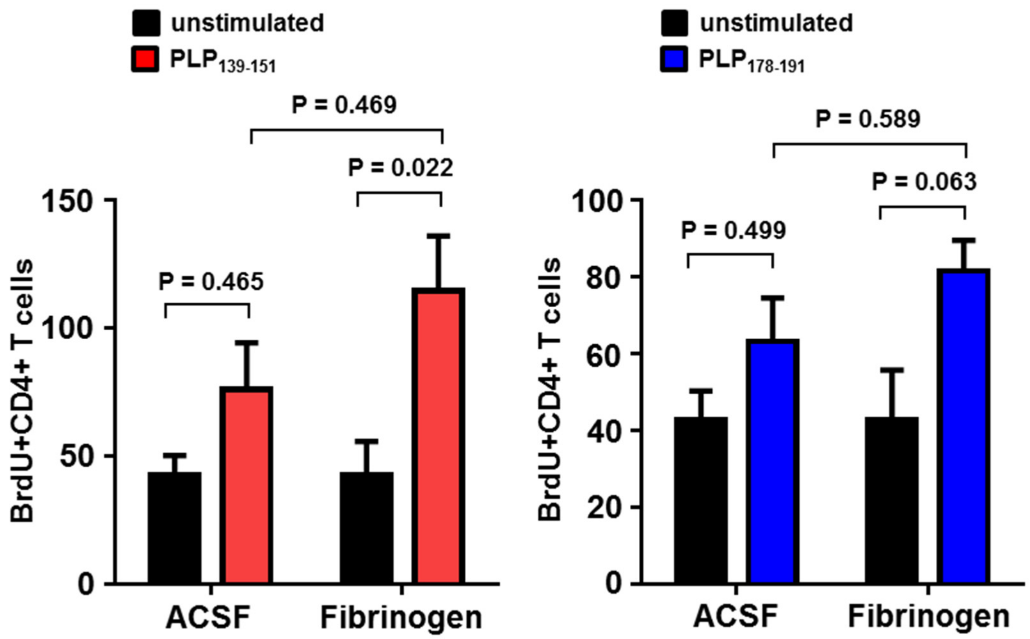

Supplementary Figure 10. CD4 T cell proliferation in response to PLP peptides in the draining lymph node in SJL/J mice injected with fibrinogen. Lymphocytes were prepared from the draining lymph nodes of fibrinogen and ACSF injected SJL/J mice at 7 days post-injection. Cells were stimulated with 20 μg/ml $PLP_{139-151}$ and 20 μg/ml $PLP_{178-191}$ for 7 days. Proliferation of $CD4^+$ T cells was measured by BrdU incorporation during the last 24 h of culture. Data are presented as mean ± s.e.m. (*n* =4 independent experiments with pooled cells from 2-3 mice per experiment for ACSF and fibrinogen, two-way ANOVA and Turkey's multiple comparison test).

Supplementary Figure 11

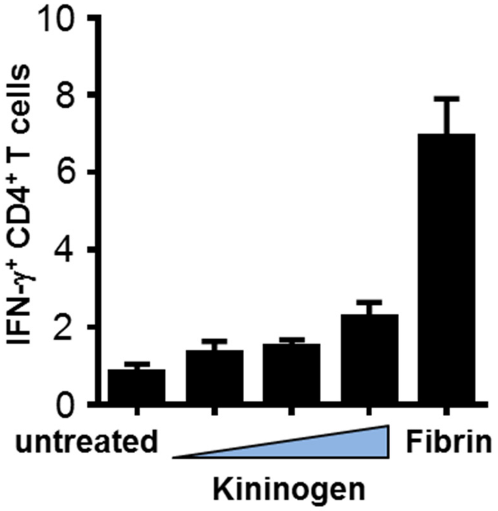

Supplementary Figure 11. Plasma protein kininogen does not mediate Th1 differentiation. Naïve $CD4^+$ T cells were co-cultured for 4 d with BMDMs that has been stimulated with different concentrations of kininogen (0.1, 0.2, and 1 ug/ml) or fibrin. Th1 cell differentiation was assessed by flow cytometry of $CD4^+$ T cells stained with IFN-γ. Treatment of kininogen does not induce Th1 differentiation as compared with fibrin. Results are from three independent experiments. Data are presented as mean ± s.e.m.

Supplementary Table 1: Genome-wide analysis of fibrinogen-injected corpus callosum analyzed for Biological Process, Molecular Function, and Cellular Component

Mouse tissue GO Name -Biological Process	Number of genes differentially expressed that were in this pathway/category	Number of genes in this pathway that were on the chip	Number of genes in this category, total	Percent Changed	Percent Present	P-value	Genes that were differentially expressed *and* are in this category
neutrophil chemotaxis(GO:0030593)	4	24	25	16.67	96.00	0	C5ar1|Ccl2|Ccl3|Fcer1g
positive regulation of leukocyte chemotaxis(GO:0002690)	4	29	29	13.79	100.00	0	C5ar1|Ccl2|Cxcl10|Figf
negative regulation of immune effector process(GO:0002698)	4	49	52	8.16	94.23	0	A2m|Hmox1|Ifit1|Serping1
immune response(GO:0006955)	17	301	317	5.65	94.95	0	C4b|Ccl12|Ccl2|Ccl3|Ccl7|Ccl9|Cd14|Cxcl10|Cxcl5 |Fcer1g|Ifnb1|Il1r1|Lcn2|Ly86|Mx2|Prg4|Serping1
response to wounding(GO:0009611)	14	266	272	5.26	97.79	0	C4b|Ccl12|Ccl2|Ccl3|Ccl7|Cd14|Cd44|Cxcl10|Cxcl5| Gfap|Hmox1|Lcp1|Ly86|Serpina3n
immune effector process(GO:0002252)	7	145	157	4.83	92.36	0	C4b|Cxcl10|Fcer1g|Ifnb1|Lcp1|Ptx3|Serping1
defense response(GO:0006952)	19	435	480	4.37	90.63	0	C4b|C5ar1|Ccl12|Ccl2|Ccl3|Ccl7|Cd14|Cxcl10|Cxcl5 | Fcer1g|Gbp2|Ifnb1|Il1r1|Lcn2|Ly86|Mx2|Npy2r| Serpina3n|Serping1
response to biotic stimulus(GO:0009607)	14	369	412	3.79	89.56	0	C5ar1|Ccl2|Cd14|Cxcl10|Fcer1g|Gbp2|Ifit1|Ifitm1| Ifitm2|Ifitm3|Ifnb1|Lcn2|Mx2|Ptx3
negative regulation of endopeptidase activity(GO:0010951)	5	138	151	3.62	91.39	0	A2m|C4b|Serpina1c|Serpina3n|Serping1
response to cytokine stimulus(GO:0034097)	7	202	209	3.47	96.65	0	Ccl2|Gbp2|Ifit1|Il1r1|Osmr|Serpina1c|Serpina3n
regulation of cytokine production(GO:0001817)	6	247	256	2.43	96.48	0	C5ar1|Ccl2|Cd14|Fcer1g|Figf|Hmox1
cation homeostasis(GO:0055080)	7	300	301	2.33	99.67	0	C5ar1|Ccl2|Ccl3|Hmox1|Mt2|Npy2r|Sgk
regulation of immune response(GO:0050776)	7	321	337	2.18	95.25	0	A2m|C4b|C5ar1|Cd44|Fcer1g|Hmox1|Serping1
regulation of angiogenesis(GO:0045765)	4	104	105	3.85	99.05	0.0005	C5ar1|Ccl2|Cxcl10|Hmox1
regulation of acute inflammatory response(GO:0002673)	3	38	44	7.89	86.36	0.0015	A2m|Fcer1g|Serping1
negative regulation of defense response(GO:0031348)	3	61	64	4.92	95.31	0.0015	A2m|Ifit1|Serping1
response to hydrogen peroxide(GO:0042542)	3	47	47	6.38	100.00	0.002	Hba-a2|Hmox1|Lcn2
positive regulation of developmental process(GO:0051094)	7	507	509	1.38	99.61	0.0025	Adamts9|C5ar1|Ccl3|Gfap|Hmox1|Msr1|Sgk
regulation of proteolysis(GO:0030162)	4	154	167	2.60	92.22	0.0035	A2m|Serpina1c|Serpina3n|Serping1
cellular response to oxidative stress(GO:0034599)	3	60	60	5.00	100.00	0.0045	Hba-a2|Hmox1|Lcn2
positive regulation of immune effector process(GO:0002699)	3	88	98	3.41	89.80	0.007	Ccl2|Fcer1g|Hmox1
regulation of neurological system process(GO:0031644)	4	212	214	1.89	99.07	0.0105	Ccl2|Gfap|Hba-a2|Npy2r
regulation of cell projection organization(GO:0031344)	4	201	202	1.99	99.50	0.0115	Gfap|Lcn2|Sgk|Vim
positive regulation of transport(GO:0051050)	5	349	357	1.43	97.76	0.014	Cd14|Fcer1g|Npy2r|Ptx3|Sgk
response to peptide hormone stimulus(GO:0043434)	3	161	166	1.86	96.99	0.0245	Serpina1c|Serpina3n|Sgk
regulation of cell death(GO:0010941)	9	963	974	0.93	98.87	0.0255	C5ar1|Ccl2|Cd44|Fcer1g|Hba-a2|Hmox1|Ifnb1|Lcn2|Sgk
regulation of vesicle-mediated transport(GO:0060627)	3	162	169	1.85	95.86	0.029	Fcer1g|Hmox1|Ptx3
positive regulation of cell proliferation(GO:0008284)	6	508	516	1.18	98.45	0.029	Ccl2|Cxcl10|Figf|Gfap|Hmox1|Osmr
regulation of cellular localization(GO:0060341)	5	418	421	1.20	99.29	0.0345	Cd14|Fcer1g|Hmox1|Lcp1|Npy2r

Mouse tissues ; GO Name – Molecular Function	Number of genes differentially expressed that were in this pathway/category	Number of genes in this pathway that were on the chip	Number of genes in this category, total	Percent Changed	Percent Present	P-value	Genes that were differentially expressed *and* are in this category
CCR2 chemokine receptor binding(GO:0031727)	3	4	4	75.0	100.0	0.000	Ccl12|Ccl2|Ccl7
chemokine activity(GO:0008009)	7	32	34	21.9	94.1	0.000	Ccl12|Ccl2|Ccl3|Ccl7|Ccl9|Cxcl10|Cxcl5
endopeptidase inhibitor activity(GO:0004866)	5	145	162	3.4	89.5	0.000	A2m|C4b|Serpina1c|Serpina3n|Serping1
pattern binding(GO:0001871)	5	156	157	3.2	99.4	0.001	Ccl2|Ccl7|Cd44|Prg4|Ptx3
cytokine binding(GO:0019955)	4	102	102	3.9	100.0	0.002	A2m|Cd44|Il1r1|Osmr
growth factor binding(GO:0019838)	3	106	106	2.8	100.0	0.011	A2m|Il1r1|Osmr
transmembrane receptor activity(GO:0004888)	13	1669	1995	0.8	83.7	0.012	C5ar1|Cd44|Fcer1g|Gpr84|Il1r1|Msr1|Npy2r|Olfr105|Olfr1163|Olfr187|Olfr521|Osmr|Prg4
protein homodimerization activity(GO:0042803)	5	465	473	1.1	98.3	0.044	Cebpd|Figf|Hmox1|Lcn2|Olfml2b
iron ion binding(GO:0005506)	3	190	197	1.6	96.4	0.047	Hba-a2|Hmox1|Lcn2

Mouse tissues; GO Name – Cellular Component	Number of genes differentially expressed that were in this pathway/category	Number of genes in this pathway that were on the chip	Number of genes in this category, total	Percent Changed	Percent Present	P-value	Genes that were differentially expressed *and* are in this category
extracellular region part(GO:0044421)	20	927	970	2.16	95.57	0.0000	A2m|Adamts9|C4b|Ccl12|Ccl2|Ccl3|Ccl7|Ccl9|Cd14|Cxcl10|Cxcl5|Figf|Gbp2|Hspa8|Ifnb1|Lcn2|Msr1|Olfml2b|Serping1|Ttr
extracellular region(GO:0005576)	23	1501	1558	1.53	96.34	0.0000	A2m|C4a|C4b|Ccl12|Ccl2|Ccl3|Ccl7|Ccl9|Cd14|Ctla2a|Cxcl10|Cxcl5|Figf|Ifnb1|Lcn2|Ly86|Olfml2b|Pla2g2c|Prg4|Ptx3|Serpina1c|Serpina3n|Serping1|Ttr
plasma membrane(GO:0005886)	23	2705	2797	0.85	96.71	0.0015	Art2b|C5ar1|Cd14|Cd44|Dsg1b|Dsp|Fcer1g|Gbp2|Gpr84|Ifitm1|Ifitm2|Ifitm3|Il1r1|Iyd|Lcp1|Lilrb4|Ly6a|Msr1|Npy2r|Olfr187|S3-12|Slc14a1|Tagln2
cell surface(GO:0009986)	6	307	309	1.95	99.35	0.0030	C5ar1|Cd14|Cd44|Cd53|Hspa8|Il1r1
external side of plasma membrane(GO:0009897)	4	192	214	2.08	89.72	0.0050	Cd44|Cxcl10|Fcer1g|Ly6a
anchored to membrane(GO:0031225)	3	130	132	2.31	98.48	0.0205	Art2b|Cd14|Ly6a
cell fraction(GO:0000267)	10	1113	1173	0.90	94.88	0.0210	Ccl3|Cyp2b13|Dsp|Gfap|Hba-a2|Hmox1|Hspa8|Iyd|Npy2r|Serpina3n
membrane raft(GO:0045121)	3	183	183	1.64	100.00	0.0385	Cd14|Fcer1g|Hmox1

Supplementary Table 2: Genome-wide analysis of fibrin-stimulated rat primary microglia analyzed for Biological Process, Molecular Function, and Cellular Component

<table>
<tr><th>Rat microglia;
GO Name – Biological Process</th><th>Number of genes differentially expressed that were in this pathway/category</th><th>Number of genes in this pathway that were on the chip</th><th>Number of genes in this category, total</th><th>Percent Changed</th><th>Percent Present</th><th>P-value</th><th>Genes that were differentially expressed *and* are in this category</th></tr>
<tr><td>immune response(GO:0006955)</td><td>66</td><td>301</td><td>346</td><td>21.9</td><td>87.0</td><td>0.0000</td><td>Bcl3|Bst2|C3|Ccl12|Ccl20|Ccl3|Ccl4|Ccl5|Ccl7|Ccl9|Cd14|Cd69|Clec4d|Csf2|Csf3|Cxcl1|Cxcl10|Cxcl11|Cxcl13|Cxcl2|Cxcl3|Cxcl9|Dlg1|Fas|Fcgr2b|Fcgr3a|H28|Il10|Il18bp|Il1a|Il1b|Il1rap|Il1rn|Il2rg|Il6|Irf7|Irgm|LOC679873|Lcn2|Lst1|Ltb|Nfkb2|Nod2|Oas1a|Oas1i|Oas2|Oasl|Oasl2|P2ry14|Plscr1|Prkcd|RT1-CE13|RT1-M6-1|RT1-N1|RT1-T24-1|Slc11a1|Sp110|Tbkbp1|Tlr10|Tlr2|Tlr7|Tnf|Tnfrsf1b|Tnfsf10|Tnfsf13|Tnfsf15</td></tr>
<tr><td>response to biotic stimulus(GO:0009607)</td><td>74</td><td>441</td><td>473</td><td>16.8</td><td>93.2</td><td>0.0000</td><td>Aars|Anxa3|Ass1|Bcl3|Bst2|Camp|Casp8|Ccl5|Cd14|Cd40|Cebpb|Cmpk2|Csf2rb|Cxcl10|Cxcl2|Cxcl9|Ddit3|Ero1l|Fas|Fcgr2b|Fgr|Gch1|H2-M3|Hck|Icam1|Ifit1|Ifit2|Ifitm3|Ifitm7|Il10|Il18bp|Il1a|Il1b|Il1rn|Il6|Irak2|Irak3|Irg1|Itgax|Klrk1|LOC360228|Lcn2|Mmp9|Mx1|Ncf1|Nfkb1|Nfkb2|Nfkbia|Nlrc4|Nlrp3|Nod2|Nos2|Nr1h3|Oas1a|Oas1b|P2ry2|Plscr1|Ppp1r15a|Prdx3|Ptges|Ptgs2|RGD1310358|Rbpj|Rsad2|Samhd1|Slc11a1|Socs3|Sod2|Sp110|Stat1|Tlr2|Tlr7|Tnf|Zc3hav1</td></tr>
<tr><td>defense response(GO:0006952)</td><td>63</td><td>388</td><td>427</td><td>16.2</td><td>90.9</td><td>0.0000</td><td>Anxa3|Ass1|Bcl3|Bst2|C3|Camp|Casp4|Ccl20|Ccl3|Ccl4|Ccl5|Ccl7|Cd14|Cd40|Cd44|Cd69|Clec4d|Cxcl1|Cxcl10|Cxcl2|Cxcl3|Cxcl9|Fcgr2b|Fgr|H2-M3|Hck|Il10|Il1a|Il1b|Il1rap|Il1rn|Il6|Irgm|Itgax|Klrk1|Kng1|Lcn2|Mefv|Ncf1|Nfkbiz|Nlrc4|Nlrp3|Nod2|Nos2|Nppb|Nupr1|Ptges|Ptgs2|RGD1559914|Rbpj|Rsad2|Samhd1|Sdc1|Slc11a1|Sp110|Tap1|Tapbp|Tbkbp1|Tlr10|Tlr2|Tlr7|Tnf|Tnfrsf1b</td></tr>
<tr><td>response to tumor necrosis factor(GO:0034612)</td><td>18</td><td>53</td><td>58</td><td>34.0</td><td>91.4</td><td>0.0000</td><td>Abcb1b|Ass1|Camp|Casp8|Ccl5|Chi3l1|Cxcl16|Fas|Gch1|Icam1|Il18bp|LOC360228|Lcn2|Mmp9|Nos2|Tnf|Tnfrsf1b|Ubd</td></tr>
<tr><td>positive regulation of nitric oxide biosynthetic process(GO:0045429)</td><td>11</td><td>24</td><td>24</td><td>45.8</td><td>100.0</td><td>0.0000</td><td>H2-M3|Icam1|Il1b|Il6|Itgb2|Jak2|Klrk1|Ptgs2|Sod2|Tlr2|Tnf</td></tr>
<tr><td>cellular response to cytokine stimulus(GO:0071345)</td><td>28</td><td>132</td><td>140</td><td>21.2</td><td>94.3</td><td>0.0000</td><td>Abcb1b|Acsl1|Ass1|Camp|Ccl5|Chi3l1|Cish|Cpd|Ebi3|Fas|Icam1|Ifit1|Ifit2|Il18bp|Il1a|Il1b|Il1rap|Il6|Irak2|Irak3|Jak2|Lcn2|Met|Mmp9|P2ry2|Stat1|Tnf|Tnfrsf1b</td></tr>
<tr><td>production of molecular mediator of immune response(GO:0002440)</td><td>6</td><td>9</td><td>9</td><td>66.7</td><td>100.0</td><td>0.0000</td><td>Dlg1|Fas|Il7r|Nod2|Slc11a1|Tnfsf13</td></tr>
<tr><td>immune effector process(GO:0002252)</td><td>27</td><td>138</td><td>145</td><td>19.6</td><td>95.2</td><td>0.0000</td><td>Anxa3|Bcl3|Bst2|C3|Cd40|Cd69|Cxcl10|Cxcl9|Dlg1|Fas|Fcgr2b|H2-M3|Icam1|Il6|Irf7|Itgal|Itgax|Ncf1|Nlrp3|Nod2|Prkcd|Rsad2|Samhd1|Slc11a1|Tlr2|Tlr7|Tnfsf13</td></tr>
<tr><td>regulation of immune system process(GO:0002682)</td><td>62</td><td>518</td><td>548</td><td>12.0</td><td>94.5</td><td>0.0000</td><td>Adora2a|C3|Ccl3|Ccl5|Cd274|Cd38|Cd40|Cd44|Cd69|Csf1|Csf2|Cxcl10|Dlg1|Fas|Fcgr2b|H2-M3|Icam1|Ifit1|Ikbke|Il10|Il1b|Il2rg|Il6|Il7r|Irak2|Irak3|Irf7|Itgal|Jag1|Klrk1|Kng1|Lst1|Lyn|Mapkapk2|Mitf|Mmp9|Nfkb1|Nfkb2|Nfkbia|Nfkbib|Niacr1|Nod2|Nr1h3|PVR|Pml|Pram1|Ptprj|Pvrl2|Samhd1|Scin|Slamf7|Slc11a1|Slc7a2|Tap1|Tlr2|Tlr5|Tlr7|Tnf|Tnfrsf14|Tnfsf13|Tnfsf13b|Zc3hav1</td></tr>
<tr><td>amino acid activation(GO:0043038)</td><td>13</td><td>43</td><td>45</td><td>30.2</td><td>95.6</td><td>0.0000</td><td>Aars|Cars|Eprs|Farsb|Gars|Lars|Mars|Nars|Sars|Tars|Vars|Wars|Yars</td></tr>
<tr><td>ISG15-protein conjugation(GO:0032020)</td><td>4</td><td>5</td><td>5</td><td>80.0</td><td>100.0</td><td>0.0000</td><td>G1p2|Uba7|Ube2l6|Usp18</td></tr>
<tr><td>regulation of interleukin-18 production(GO:0032661)</td><td>3</td><td>3</td><td>4</td><td>100.0</td><td>75.0</td><td>0.0000</td><td>Nod2|Tlr2|Tnf</td></tr>
<tr><td>cadmium ion transport(GO:0015691)</td><td>3</td><td>3</td><td>3</td><td>100.0</td><td>100.0</td><td>0.0000</td><td>Slc11a1|Slc11a2|Slc30a1</td></tr>
<tr><td>regulation of defense response(GO:0031347)</td><td>36</td><td>251</td><td>261</td><td>14.3</td><td>96.2</td><td>0.0000</td><td>Adora2a|Aoah|C3|Ccl5|Fcgr2b|H2-M3|Ifit1|Ikbke|Il10|Il1b|Il6|Irak2|Irak3|Irf7|Jak2|Klrk1|Mapkapk2|Nfkb1|Nfkb2|Nfkbia|Nfkbib|Nod2|PVR|Pik3cg|Pml|Ptgs2|Pvrl2|Samhd1|Slc7a2|Tap1|Tgm2|Tlr2|Tlr7|Tnf|Tnfrsf1b|Zc3hav1</td></tr>
<tr><td>regulation of apoptosis(GO:0042981)</td><td>83</td><td>862</td><td>908</td><td>9.6</td><td>94.9</td><td>0.0000</td><td>Aars|Actn4|Adar|Adora2a|Angpt1|Arhgef3|Asns|Atf5|Bcl2a1d|Bcl3|Birc2|Birc3|Btg2|Casp4|Casp7|Casp8|Ccl5|Cd38|Cd40|Cd44|Cebpb|Cflar|Csf2|Daxx|Ddit3|Fas|Frag1|Gch1|Ifi27l|Ifih1|Ikbke|Il10|Il1b|Il1rn|Il6|Itgb1|Jak2|Lcn2|MGC94600|Mitf|Mmp9|Myc|Naip2|Nfkb1|Nfkbia|Niacr1|Nlrc4|Nlrp3|Nod2|Nos2|Nqo1|Nr3c1|Nup62|Nupr1|Pik3cg|Pim1|Plagl2|Pml|Prdx3|Prex1|Prkcd|Ptgs2|Ptprj|Rarg|Rbpj|Ripk3|Scin|Sfrp2|Skil|Slc11a2|Sod2|Sp110|Sqstm1|Stat1|Steap3|Sycp3|Tgm2|Tnf|Tnfsf10|Tnfsf13b|Tnfsf15|Trim35|Ubd</td></tr>
<tr><td>negative regulation of viral reproduction(GO:0048525)</td><td>4</td><td>6</td><td>6</td><td>66.7</td><td>100.0</td><td>0.0000</td><td>Ccl5|Ifit1|Oas1a|Tnf</td></tr>
<tr><td>negative regulation of interleukin-12 production(GO:0032695)</td><td>4</td><td>6</td><td>8</td><td>66.7</td><td>75.0</td><td>0.0000</td><td>Il10|Irak3|Nod2|Tlr2</td></tr>
<tr><td>positive regulation of cytokine production(GO:0001819)</td><td>21</td><td>120</td><td>128</td><td>17.5</td><td>93.8</td><td>0.0000</td><td>Bcl3|C3|Cd14|Cd40|Csf2|H2-M3|Il1a|Il1b|Il6|Jak2|Klrk1|Nlrp3|Nod2|P2ry2|Ptgs2|Slc11a1|Tlr2|Tlr5|Tlr7|Tnf|Zc3hav1</td></tr>
<tr><td>antigen processing and presentation(GO:0019882)</td><td>14</td><td>62</td><td>90</td><td>22.6</td><td>68.9</td><td>0.0000</td><td>Fcgr2b|H2-M3|Icam1|LOC679873|Psmb8|Psmb9|Psme2|RT1-CE13|RT1-M6-1|RT1-N1|RT1-T24-1|Slc11a1|Tap1|Tapbp</td></tr>
<tr><td>L-serine biosynthetic process(GO:0006564)</td><td>3</td><td>4</td><td>4</td><td>75.0</td><td>100.0</td><td>0.0000</td><td>Phgdh|Psph|Shmt2</td></tr>
</table>

regulation of NF-kappaB import into nucleus(GO:0042345)	8	25	26	32.0	96.2	0.0000	Bcl3|Il1b|Nfkbia|Nfkbib|Nlrp3|Tlr2|Tlr7|Tnf
response to exogenous dsRNA(GO:0043330)	6	15	16	40.0	93.8	0.0000	Ifit1|Irak3|Nfkbia|Nod2|Stat1|Zc3hav1
myeloid leukocyte activation(GO:0002274)	12	52	55	23.1	94.5	0.0000	Anxa3|Ccl5|Csf2|Fcgr2b|Fyb|Nod2|Rhoh|Slc11a1|Slc7a2|Tlr2|Tlr7|Ubd
protein secretion(GO:0009306)	8	27	31	29.6	87.1	0.0000	Cxcl10|Lyn|Nlrc4|Nlrp3|Plek|Pou2f2|Steap3|Tnfsf13b
regulation of vascular endothelial growth factor production(GO:0010574)	5	12	14	41.7	85.7	0.0000	C3|Il1a|Il1b|Il6|Ptgs2
negative regulation of vascular permeability(GO:0043116)	4	8	8	50.0	100.0	0.0000	Adora2a|Angpt1|Nr3c1|Ptprj
regulation of I-kappaB kinase/NF-kappaB cascade(GO:0043122)	19	119	122	16.0	97.5	0.0000	Birc2|Bst2|Casp8|Cd40|Cflar|Ikbke|Il1a|Il1b|Lgals9|MGC94600|Nek6|Nod2|Nup62|Rhoh|Sqstm1|Tgm2|Tnf|Tnfsf10|Ubd
neutrophil chemotaxis(GO:0030593)	7	23	24	30.4	95.8	0.0000	Ccl3|Cklf|Cxcl1|Cxcl2|Cxcl3|Il1b|Itgb2
manganese ion transmembrane transport(GO:0071421)	3	5	5	60.0	100.0	0.0005	Slc11a1|Slc11a2|Trpm2
arginine transport(GO:0015809)	3	5	5	60.0	100.0	0.0005	Slc11a1|Slc7a1|Slc7a2
positive regulation of sequence-specific DNA binding transcription factor activity(GO:0051091)	16	93	97	17.2	95.9	0.0000	Anxa3|Fzd1|Hmgn3|Icam1|Il10|Il1b|Il6|Irak2|Irak3|Itgb2|Jak2|Nod2|Ppap2b|Prdx3|Tlr2|Tnf
regulation of protein metabolic process(GO:0051246)	76	909	947	8.4	96.0	0.0000	Acsl1|Adora2a|Angpt1|Bcl3|C3|Camkk2|Camp|Ccl5|Cd44|Ceacam1|Cebpb|Cish|Csf1|Csf2|Daxx|Dlg1|Dusp16|Eif2b3|Eif4ebp1|Fam129a|Fzd1|Hcls1|Icoslg|Il10|Il1a|Il1b|Il1rn|Il6|Irak2|Irak3|Itga5|Itgb1|Itgb2|Jak2|Kng1|LOC682999|LOC683722|Ltb|Lyn|Met|Mknk1|N4bp1|Nfkb1|Nfkbia|Nod2|Nr1h3|Nup62|Pim1|Pion|Piwil2|Pkia|Pml|Ppap2b|Ppp1r15a|Prkcd|Psmb10|Psmb8|Psmb9|Psme2|Ptk2b|Ptprj|RGD1561067|Sdc4|Sfrp2|Slc11a1|Socs3|Spry1|Sqstm1|Sv2b|Tlr2|Tlr7|Tnf|Tnfrsf14|Tnfsf15|Trib3|Trim21
regulation of interleukin-6 production(GO:0032675)	10	48	51	20.8	94.1	0.0000	Cebpb|Il10|Il1a|Il1b|Il6|Irak3|Nod2|Tlr2|Tlr7|Tnf
regulation of cytokine secretion(GO:0050707)	9	41	42	22.0	97.6	0.0000	Cd14|Il10|Il1a|Il6|Nlrp3|Nod2|Srgn|Tnf|Tnfsf15
osteoclast differentiation(GO:0030316)	5	15	16	33.3	93.8	0.0000	Cd300lf|Csf1|Junb|Mitf|Tnf
regulation of cell activation(GO:0050865)	26	217	230	12.0	94.3	0.0000	Adora2a|Ccl5|Cd274|Cd38|Cd40|Dlg1|Fas|Fcgr2b|Il10|Il1b|Il2rg|Il6|Il7r|Itgal|Jak2|Kng1|Lst1|Nod2|Plek|Pram1|Pvrl2|Slamf7|Slc7a2|Tnfrsf14|Tnfsf13|Tnfsf13b
regulation of calcidiol 1-monooxygenase activity(GO:0060558)	3	6	7	50.0	85.7	0.0005	Il1b|Nfkb1|Tnf
zinc ion transport(GO:0006829)	6	21	22	28.6	95.5	0.0000	Slc11a2|Slc30a1|Slc39a1|Slc39a13|Slc39a14|Slc39a4l|Slc39a8
response to mechanical stimulus(GO:0009612)	16	106	113	15.1	93.8	0.0000	Asns|Btg2|Cxcl10|Il6|Itgb1|Junb|Mkks|Mmp14|Mmp9|Myc|Nos2|Prkcd|Psph|Ptk2b|Stat1|Tnf
regulation of cell proliferation(GO:0042127)	68	835	870	8.1	96.0	0.0000	Adora2a|Angpt1|Atf5|Btg2|Camp|Ccl5|Cd274|Cd38|Cd40|Cdh5|Csf1|Csf2|Cxcl10|Dlg1|Egln3|Fas|Fcgr2b|Hcls1|Ifitm3|Il10|Il1a|Il1b|Il6|Itgal|Itgb1|Jag1|Jak2|Kng1|LOC683722|Lst1|Lyn|Mitf|Mmp12|Myc|Nampt|Nfkbia|Nod2|Nos2|Nppb|Nr3c1|Ntn1|Nup62|Nupr1|Pml|Prdx3|Ptger2|Ptges|Ptgs2|Ptk2b|Ptprj|Ptprm|Rac2|Rarg|Rbpj|Scin|Sfrp2|Shmt2|Slfn3|Sod2|Spry1|Stat1|Tgm2|Tnf|Tnfrsf14|Tnfsf13|Tnfsf13b|Trim35|Vash1
leukocyte cell-cell adhesion(GO:0007159)	7	28	28	25.0	100.0	0.0000	Ccl5|Icam1|Itga5|Itgal|Itgb1|Itgb2|Tnf
regulation of angiogenesis(GO:0045765)	15	98	105	15.3	93.3	0.0000	Anxa3|C3|Camp|Ccl5|Cxcl10|Il1a|Il1b|Itgb2|Mmp9|Pml|Ptgs2|Ptprm|Sfrp2|Vash1|Wars
response to heat(GO:0009408)	13	81	84	16.0	96.4	0.0000	Cd14|Cxcl10|Eif2b3|Il1a|Il1b|Il6|Lyn|Mmp9|Mthfd2|Myof|Prkcd|Socs3|Tfec
regulation of interferon-gamma production(GO:0032649)	9	45	49	20.0	91.8	0.0000	Bcl3|H2-M3|Il10|Il1b|Klrk1|Nod2|Slc11a1|Tlr7|Tnf
regulation of cellular component movement(GO:0051270)	30	287	298	10.5	96.3	0.0000	Actn1|Actn4|Angpt1|Anxa3|Ccl5|Clic4|Csf1|Cxcl10|Cxcl16|Icam1|Itga5|Itgb1|Jak2|LOC683722|Lyn|Mkks|Mmp9|Nod2|Nrp1|Ntn1|P2ry2|Ptgs2|Ptk2b|Ptprj|Ptprm|Rbpj|Rras|Sfrp2|Tlr2|Vash1
response to organic nitrogen(GO:0010243)	19	154	157	12.3	98.1	0.0000	Aars|Adora2a|Ak3l1|Asns|Ass1|Birc2|Btg2|Ddit3|Icam1|Il1a|Il1b|Il1rn|Il6|Lyn|Map2k1ip1|Prkcd|Ptgs2|Ptk2b|Ubd
negative regulation of multi-organism process(GO:0043901)	6	25	26	24.0	96.2	0.0000	Camp|Ifit1|Il10|Nod2|Tlr2|Tnf
response to organic cyclic compound(GO:0014070)	30	298	315	10.1	94.6	0.0000	Abcb1b|Acsl1|Adora2a|Ak3l1|Asns|Birc2|Btg2|Ccl5|Cd44|Eno2|Icam1|Il1a|Il1b|Il1rn|Il6|Junb|Lyn|Met|Mmp14|Mmp9|Nampt|Nppb|P2ry2|Prkcd|Ptges|Ptgs2|Ptk2b|Slc7a11|Socs3|Stat1
programmed cell death(GO:0012501)	35	370	388	9.5	95.4	0.0000	Acp2|Arhgef3|Bcl2a1d|Casp4|Casp7|Casp8|Cd40|Chac1|Cib1|Daxx|Ece1|Egln3|Fas|Ghitm|Il6|Jak2|MGC94600|Myc|Ncf1|Nek6|Niacr1|Ppp1r15a|Prex1|Ripk3|Sfrp2|Sod2|Sp110|Sqstm1|Sra1|Srgn|Steap3|Tnf|Trib3|Trim69|Zc3h12a
modulation of growth of symbiont involved in interaction with host(GO:0044144)	5	19	19	26.3	100.0	0.0005	Camp|Il10|Nod2|Tlr2|Tnf
regulation of growth of symbiont in host(GO:0044126)	5	19	19	26.3	100.0	0.0005	Camp|Il10|Nod2|Tlr2|Tnf

regulation of locomotion(GO:0040012)	29	290	302	10.0	96.0	0.0000	Adora2a|Angpt1|Anxa3|Ccl5|Clic4|Csf1|Cxcl10|Cxcl16|Cxcl9|Icam1|Itga5|Itgb1|Jak2|LOC683722|Mkks|Mmp9|Nod2|Nrp1|Ntn1|P2ry2|Ptgs2|Ptk2b|Ptprj|Ptprm|Rbpj|Rras|Sfrp2|Tlr2|Vash1
neutral amino acid transport(GO:0015804)	6	26	27	23.1	96.3	0.0000	Nfkbie|Slc1a4|Slc1a5|Slc3a2|Slc6a9|Slc7a5
positive regulation of peptidase activity(GO:0010952)	12	80	82	15.0	97.6	0.0005	Casp4|Ifi27l|Mmp14|Myc|Nlrc4|Nlrp3|Pml|Psme2|Sfrp2|Slc11a2|Tnf|Tnfsf15
response to hypoxia(GO:0001666)	24	231	240	10.4	96.3	0.0000	Abcb1b|Actn4|Agtrap|Angpt1|Birc2|Cd38|Ece1|Egln3|Fas|Icam1|Il1a|Il1b|Mmp14|Mmp9|Nos2|Nppb|Pml|Prkcd|Ptk2b|Slc11a2|Socs3|Sod2|Tlr2|Tnf
response to drug(GO:0042493)	37	419	443	8.8	94.6	0.0000	Abcb1b|Acsl1|Adora2a|Ass1|Ccl3|Ccl5|Cd38|Ddit3|Eno2|Fas|Fzd1|Gk|Gsta3|Havcr1|Icam1|Il10|Il1b|Il1rn|Il6|Itgb1|Junb|Lcn2|Lyn|Met|Mmp12|Mmp9|Myc|Nppb|Prkcd|Ptgs2|Ptk2b|Ptprm|Sfrp2|Socs3|Sod2|Stat1|Tnf
regulation of chemokine production(GO:0032642)	6	28	29	21.4	96.6	0.0000	Il1b|Il6|P2ry2|Tlr2|Tlr7|Tnf
response to hormone stimulus(GO:0009725)	53	689	729	7.7	94.5	0.0000	Abcb1b|Angpt1|Anxa3|Asns|Ass1|Bckdhb|Btg2|Casp8|Ccl5|Cd38|Eif2b3|Eif4ebp1|Eno2|Fas|Fzd1|Gcgr|Hcls1|Hsd11b1|Icam1|Ifi27l|Il10|Il1b|Il1rn|Il6|Insig1|Itgb1|Jak2|Junb|Lyn|Me1|Met|Mmp14|Mmp9|Nampt|Nos2|Nppb|Nr1h3|Nr3c1|Prkcd|Psph|Ptgs2|Ptk2b|Ptpn1|Retn|Rhoq|Sdc1|Slc25a36|Socs3|Stat1|Tlr2|Tnf|Tnfsf10|Trib3
integrin-mediated signaling pathway(GO:0007229)	8	47	50	17.0	94.0	0.0000	Cib1|Itga5|Itgal|Itgax|Itgb1|Itgb2|Plek|Pram1
regulation of cell killing(GO:0031341)	7	38	41	18.4	92.7	0.0005	H2-M3|Il7r|Klrk1|Nos2|PVR|Pvrl2|Tap1
epithelial cell proliferation(GO:0050673)	7	39	40	17.9	97.5	0.0005	Cebpb|Csf2|Dlg1|Il6|Mmp14|Nos2|Tnf
aging(GO:0007568)	20	194	206	10.3	94.2	0.0000	Ass1|Casp7|Ccl5|Ddit3|Fas|Gsta3|Icam1|Il1b|Il6|Met|Mmp9|Nfkb2|Nos2|Nr3c1|Nup62|P2ry2|Pml|Prkcd|Socs3|Sod2
cellular response to protein stimulus(GO:0071445)	10	72	76	13.9	94.7	0.0000	Aars|Ccl5|Cd44|Ddit3|Ero1l|Itgb1|Lcn2|Myc|Nrp2|Ppp1r15a
neuron projection development(GO:0031175)	12	101	104	11.9	97.1	0.0000	Btg2|Cd44|Il6|Itgb1|Lst1|Lyn|Nrp1|Phgdh|Ptk2b|Ptprm|Scarf1|Stx3
response to hydroperoxide(GO:0033194)	4	9	9	44.4	100.0	0.0010	Ccl5|Cd38|Jak2|Trpm2
iron ion transmembrane transport(GO:0034755)	3	7	7	42.9	100.0	0.0010	Slc11a2|Slc25a37|Slc39a14
response to vitamin D(GO:0033280)	6	28	32	21.4	87.5	0.0010	Cxcl10|Fzd1|Il1b|Itgb1|Nos2|Ptgs2
response to endoplasmic reticulum stress(GO:0034976)	6	35	38	17.1	92.1	0.0010	Aars|Atf4|Ddit3|Ero1l|Fam129a|Ppp1r15a
'de novo' protein folding(GO:0006458)	5	18	18	27.8	100.0	0.0015	Ero1l|RGD1308019|Sep15|Tor1b|Tor3a
regulation of cell-substrate junction assembly(GO:0090109)	3	8	9	37.5	88.9	0.0015	Mmp14|Ptprj|Sdc4
negative regulation of NF-kappaB transcription factor activity(GO:0032088)	6	33	35	18.2	94.3	0.0015	Irak2|Irak3|Nfkbia|Nlrp3|Nod2|Trim21
intracellular receptor mediated signaling pathway(GO:0030522)	8	61	62	13.1	98.4	0.0015	Daxx|Jak2|Nfkbia|Nod2|Nos2|Nr3c1|Pml|Rarg
cellular response to glucose starvation(GO:0042149)	3	8	8	37.5	100.0	0.0020	Asns|Prkcd|Slc2a1
response to interferon-gamma(GO:0034341)	6	28	29	21.4	96.6	0.0020	Ass1|Ccl5|Cxcl16|Gch1|Slc11a1|Ubd
protein import into nucleus, translocation(GO:0000060)	4	16	17	25.0	94.1	0.0020	Bcl3|Nfkbia|Slc11a1|Tnf
positive regulation of cell adhesion(GO:0045785)	10	84	88	11.9	95.5	0.0020	Angpt1|Ccl5|Csf1|Itga5|Itgal|Itgb1|Ptprj|Sdc4|Tgm2|Tnf
cell redox homeostasis(GO:0045454)	8	61	67	13.1	91.0	0.0020	Ddit3|Gsr|Il6|Pdia5|Prdx3|Ptges2|Sco1|Txnrd1
wound healing(GO:0042060)	9	78	83	11.5	94.0	0.0020	Cd44|Fcgr3a|Il1a|Il1b|Nos2|P2ry2|Sdc1|Serpinb2|Slc11a1
response to hydrogen peroxide(GO:0042542)	10	95	99	10.5	96.0	0.0020	Ddit3|Il18bp|Lcn2|Prdx3|Prkcd|Ptk2b|Sdc1|Sod2|Stat1|Txnrd1
L-amino acid transport(GO:0015807)	6	34	34	17.6	100.0	0.0025	Slc11a1|Slc1a4|Slc1a5|Slc7a1|Slc7a2|Slc7a5
response to gamma radiation(GO:0010332)	6	35	36	17.1	97.2	0.0025	Cxcl10|Il1a|Il1b|Pml|Socs3|Sod2
morphogenesis of a branching epithelium(GO:0061138)	12	118	119	10.2	99.2	0.0025	Cd44|Csf1|Dlg1|Il10|Il6|Myc|Nrp1|Plxnd1|Pml|Sfrp2|Socs3|Tgm2
cell adhesion mediated by integrin(GO:0033627)	3	8	8	37.5	100.0	0.0030	Icam1|Itga5|Itgb1
regulation of glutamate secretion(GO:0014048)	4	19	19	21.1	100.0	0.0030	Adora2a|Il1b|Il1rn|Nr3c1
regulation of glucose transport(GO:0010827)	6	42	44	14.3	95.5	0.0030	Il1b|Met|Prkcd|Rhoq|Tnf|Trib3
cellular response to oxidative stress(GO:0034599)	9	77	80	11.7	96.3	0.0030	Ccl5|Il18bp|Lcn2|Pml|Prdx3|Slc11a2|Sod2|Stx3|Txnrd1
protein autophosphorylation(GO:0046777)	10	92	96	10.9	95.8	0.0030	Camkk2|Csnk1g3|Irak2|Irak3|Jak2|Lmtk2|Lyn|Met|Pim1|Prkcd
dicarboxylic acid transport(GO:0006835)	4	16	17	25.0	94.1	0.0035	Slc13a3|Slc1a4|Slc1a5|Slc25a10
lymph node development(GO:0048535)	3	11	11	27.3	100.0	0.0035	Cxcl13|Il7r|Ltb
positive regulation of ERK1 and ERK2 cascade(GO:0070374)	6	41	43	14.6	95.3	0.0035	Angpt1|Cd44|Il1a|Il1b|Il6|Nod2
interaction with symbiont(GO:0051702)	4	19	20	21.1	95.0	0.0040	Camp|Icam1|Ncf1|Pvrl2
activated T cell proliferation(GO:0050798)	3	9	9	33.3	100.0	0.0045	Itgal|Itgax|Itgb2
oligodendrocyte development(GO:0014003)	4	20	20	20.0	100.0	0.0045	Eif2b3|Lyn|Met|RGD1306622
anatomical structure maturation(GO:0071695)	3	9	10	33.3	90.0	0.0050	Cd44|Cdh5|Rbpj

positive regulation of endocytosis(GO:0045807)	7	57	59	12.3	96.6	0.0055	Actn4|Angpt1|C3|Fcgr2b|Itgb1|Nod2|Slc11a1
positive regulation of lipid transport(GO:0032370)	4	19	19	21.1	100.0	0.0060	Il1b|Nfkbia|Nr1h3|P2ry2
amino acid transmembrane transport(GO:0003333)	6	44	44	13.6	100.0	0.0060	Slc1a4|Slc1a5|Slc6a9|Slc7a1|Slc7a2|Slc7a5
response to toxin(GO:0009636)	11	115	120	9.6	95.8	0.0060	Asns|Ass1|Fas|Havcr1|Lcn2|Lyn|Nupr1|Sdc1|Slc30a1|Slc7a11|Tlr2
negative regulation of lipid catabolic process(GO:0050995)	3	12	14	25.0	85.7	0.0065	Il1b|Niacr1|Tnf
phagocytosis(GO:0006909)	5	32	33	15.6	97.0	0.0070	Anxa3|Hck|Nr1h3|Slc11a1|Tgm2
positive regulation of monooxygenase activity(GO:0032770)	3	12	12	25.0	100.0	0.0075	Gch1|Il1b|Tnf
response to manganese ion(GO:0010042)	3	12	12	25.0	100.0	0.0075	Ptgs2|Slc11a2|Sod2
pteridine-containing compound biosynthetic process(GO:0042559)	4	17	21	23.5	81.0	0.0080	Gch1|Mocs1|Mocs2|Mthfd2
positive chemotaxis(GO:0050918)	4	18	18	22.2	100.0	0.0080	Angpt1|Ccl3|Ccl5|Ptprj
nitric oxide metabolic process(GO:0046209)	3	12	12	25.0	100.0	0.0100	Nos2|Slc7a2|Tlr2
response to L-ascorbic acid(GO:0033591)	3	12	13	25.0	92.3	0.0100	Il1a|Il1b|Sod2
positive regulation of anti-apoptosis(GO:0045768)	5	33	33	15.2	100.0	0.0100	Angpt1|Btg2|Il6|Ptk2b|Sfrp2
cortical actin cytoskeleton organization(GO:0030866)	3	13	13	23.1	100.0	0.0110	Dlg1|Plek|Rhoq
positive regulation of nuclear division(GO:0051785)	4	25	25	16.0	100.0	0.0110	Il1a|Il1b|Met|Tnf
regulation of mitochondrial membrane potential(GO:0051881)	3	12	12	25.0	100.0	0.0115	Adora2a|Prdx3|Sod2
prostanoid biosynthetic process(GO:0046457)	3	13	13	23.1	100.0	0.0115	Ptges|Ptgs2|RGD1308251
bone remodeling(GO:0046849)	3	13	13	23.1	100.0	0.0125	Il6|LOC24906|Mitf
vasodilation(GO:0042311)	4	26	26	15.4	100.0	0.0130	Adora2a|Gch1|Kng1|Sod2
positive regulation of stress-activated MAPK cascade(GO:0032874)	3	12	12	25.0	100.0	0.0145	Il1a|Il1b|Nod2
response to activity(GO:0014823)	6	50	50	12.0	100.0	0.0150	Ccl5|Il10|Itgb1|Nr3c1|Sod2|Tnf
cytokine metabolic process(GO:0042107)	3	17	19	17.6	89.5	0.0155	Il1rap|Irf7|Tnfsf15
regulation of fat cell differentiation(GO:0045598)	5	40	41	12.5	97.6	0.0160	Insig1|Ptgs2|Sfrp2|Sod2|Trib3
positive regulation of cell projection organization(GO:0031346)	9	101	105	8.9	96.2	0.0165	Cdc42ep2|Itgb1|Lcn2|Met|Ntn1|P2ry2|Rhoq|Sema4d|Skil
purinergic receptor signaling pathway(GO:0035587)	3	14	14	21.4	100.0	0.0175	Adora2a|Niacr1|P2ry2
regulation of insulin receptor signaling pathway(GO:0046626)	4	25	25	16.0	100.0	0.0190	Il1b|Prkcd|Ptpn1|Socs3
hydrogen peroxide metabolic process(GO:0042743)	4	25	29	16.0	86.2	0.0190	Ncf1|Prdx3|Sod2|Txnrd1
response to growth factor stimulus(GO:0070848)	8	91	100	8.8	91.0	0.0195	Anxa3|Ccl5|Fas|Fzd1|Itgb1|Met|P2ry2|Skil
regulation of macrophage derived foam cell differentiation(GO:0010743)	3	15	16	20.0	93.8	0.0200	Csf1|Csf2|Nfkbia
response to alkaloid(GO:0043279)	9	101	104	8.9	97.1	0.0205	Adora2a|Ccl5|Icam1|Il1b|Il6|Mmp9|Myc|Ptk2b|Slc7a11
regulation of organ morphogenesis(GO:2000027)	8	87	89	9.2	97.8	0.0205	Csf1|Fzd1|Met|Myc|Sfrp2|Spry1|Stat1|Tnf
iron ion homeostasis(GO:0055072)	5	39	54	12.8	72.2	0.0210	RGD1306939|Slc11a1|Slc11a2|Sod2|Ttc7
spleen development(GO:0048536)	3	17	18	17.6	94.4	0.0215	Bcl3|Fas|Nfkb2
negative regulation of ossification(GO:0030279)	3	17	18	17.6	94.4	0.0215	P2ry2|Ptk2b|Srgn
regulation of insulin secretion(GO:0050796)	7	72	79	9.7	91.1	0.0220	Cd38|Hmgn3|Il1b|Jak2|Nos2|Pfkl|Tnf
protein monoubiquitination(GO:0006513)	3	14	14	21.4	100.0	0.0230	Dtx3l|Mgrn1|Trim21
positive regulation of JNK cascade(GO:0046330)	4	29	30	13.8	96.7	0.0240	Il1a|Il1b|Nod2|Tnf
lymphocyte homeostasis(GO:0002260)	4	28	29	14.3	96.6	0.0245	Bcl2a1d|Fas|Skil|Tnfsf13b
maintenance of location(GO:0051235)	7	68	79	10.3	86.1	0.0245	Il1b|LOC683722|Nfkbia|Nfkbib|Pml|Srgn|Tnf
negative regulation of phosphorylation(GO:0042326)	7	74	75	9.5	98.7	0.0245	Fam129a|Met|Pkia|Ppap2b|Prkcd|Rhoh|Sfrp2
glutamine family amino acid metabolic process(GO:0009064)	5	44	47	11.4	93.6	0.0255	Asns|Ass1|Nos2|Phgdh|Pycs
viral reproductive process(GO:0022415)	5	42	43	11.9	97.7	0.0260	Icam1|Ifit1|Mmp9|Pvrl2|Tap1
cellular response to inorganic substance(GO:0071241)	6	59	59	10.2	100.0	0.0260	Ass1|Clic4|Fas|Junb|Met|Nr3c1
pigment cell differentiation(GO:0050931)	3	17	17	17.6	100.0	0.0280	Mitf|Mreg|Sod2
negative regulation of tumor necrosis factor production(GO:0032720)	3	19	20	15.8	95.0	0.0295	Il10|Irak3|Nod2

positive regulation of protein kinase B signaling cascade(GO:0051897)	4	30	32	13.3	93.8	0.0300	Angpt1|F10|Il6|Ptprj
negative regulation of calcium ion transport(GO:0051926)	3	19	20	15.8	95.0	0.0310	Icam1|Ptgs2|Slc30a1
regulation of type I interferon production(GO:0032479)	3	18	19	16.7	94.7	0.0315	Tlr2|Tlr7|Zc3hav1
triglyceride biosynthetic process(GO:0019432)	4	31	32	12.9	96.9	0.0315	Acsl1|Dgat2|Gk|Lpin2
response to antibiotic(GO:0046677)	5	47	47	10.6	100.0	0.0315	Casp8|Havcr1|Il6|Jak2|Skil
protein oligomerization(GO:0051259)	18	257	270	7.0	95.2	0.0315	Angpt1|Birc2|Birc3|Casp8|Ccl5|Fas|Gch1|Lcn2|Lims1|Me1|Pfkl|Sept11|Shmt2|Skil|Sod2|Sqstm1|Tgm2|Trim21
negative regulation of G-protein coupled receptor protein signaling pathway(GO:0045744)	3	21	22	14.3	95.5	0.0330	Adrbk2|Mgrn1|Plek
acidic amino acid transport(GO:0015800)	3	18	18	16.7	100.0	0.0360	Arl6ip5|Prkcd|Slc13a3
pyrimidine nucleotide metabolic process(GO:0006220)	4	30	34	13.3	88.2	0.0345	Cmpk2|Ctps|Dck|Upp1
glutathione metabolic process(GO:0006749)	4	31	36	12.9	86.1	0.0365	Gsr|Gsta3|Gsta4|Sod2
response to cold(GO:0009409)	4	33	35	12.1	94.3	0.0385	Casp8|Cxcl10|Gk|Il6
lipoprotein metabolic process(GO:0042157)	3	22	27	13.6	81.5	0.0360	Apol3|Apol9a|RGD1309808
response to arsenic-containing substance(GO:0046685)	3	20	21	15.0	95.2	0.0395	Abcb1b|Met|Nr3c1
release of cytochrome c from mitochondria(GO:0001836)	3	20	21	15.0	95.2	0.0410	Casp7|Myc|Sod2
JAK-STAT cascade(GO:0007259)	3	18	20	16.7	90.0	0.0420	Jak2|Socs3|Stat1
response to fatty acid(GO:0070542)	4	33	34	12.1	97.1	0.0445	Acsl1|Ass1|Insig1|Tlr2
I-kappaB kinase/NF-kappaB cascade(GO:0007249)	3	19	20	15.8	95.0	0.0450	Bcl3|Ripk3|Tank
pyrimidine nucleoside metabolic process(GO:0006213)	3	21	25	14.3	84.0	0.0495	Ctps|Dck|Upp1

Rat microglia: GO Name - Molecular function	Number of genes differentially expressed that were in this pathway/category	Number of genes in this pathway that were on the chip	Number of genes in this category, total	Percent Changed	Percent Present	P-value	Adjusted P-value	Genes that were differentially expressed *and* are in this category
chemokine activity(GO:0008009)	16	33	34	48.5	97.1	0	0.0182	Ccl12|Ccl20|Ccl3|Ccl4|Ccl5|Ccl7|Ccl9|Cklf|Cxcl1|Cxcl10|Cxcl11|Cxcl13|Cxcl16|Cxcl2|Cxcl3|Cxcl9
interleukin-1 receptor binding(GO:0005149)	5	7	8	71.4	87.5	0	0.0182	Il1a|Il1b|Il1rap|Il1rn|Tlr5
aminoacyl-tRNA ligase activity(GO:0004812)	14	52	54	26.9	96.3	0	0.0182	Aars|Cars|Eprs|Farsb|Gars|Iars|Lars|Mars|Nars|Sars|Tars|Vars|Wars|Yars
2'-5'-oligoadenylate synthetase activity(GO:0001730)	3	4	4	75.0	100.0	0	0.0182	Oas1a|Oas2|Oasl2
hydrolase activity, acting on carbon-nitrogen (but not peptide) bonds, in cyclic amidines(GO:0016814)	7	24	27	29.2	88.9	0	0.0182	Adar|Ampd3|Apobec3f|Gch1|Gda|Mthfd2|Zbp1
purine ribonucleoside triphosphate binding(GO:0035639)	111	1526	1759	7.3	86.8	0	0.0182	Aars|Abcb1b|Acsl1|Adrbk2|Ak3l1|Arl5c|Asns|Ass1|Atad1|Atp11b|Atp13a1|Atp13a2|Atp8b4|Camkk2|Cars|Cmpk2|Csnk1g3|Dcakd|Dck|Ddx52|Ddx58|Ddx60|Dhx58|Entpd2|Eprs|Farsb|Fgr|Gars|Gbp2|Gbp4|Gbp5|Gch1|Gk|Gtf2f2|Hck|Hk3|Hspa9|Ifih1|Igtp|Ikbke|Irak2|Irak3|Irgm|Jak2|Kcnj1|Lars|Lmtk2|Lyn|Map3k8|Mapk6|Mapkapk2|Mark1|Mars|Mast4|Met|Mkks|Mknk1|Mov10|Mras|Mx1|Mx2|Myo1d|N4bp2l1|Nars|Nek6|Niacr1|Nlrc4|Nlrp3|Nod2|Oas1a|Oas1i|Oas2|Oasl|Oasl2|P2ry2|Pfkl|Pim1|Pric285|Prkcd|Prkch|Ptk2b|RGD1305184|RGD1306880|RGD1308019|Rab20|Rab32|Rac2|Rhoh|Rhoq|Riok3|Ripk3|Rnd1|Rras|Sars|Sept11|Slfn3|Slfn4|Slfn5|Tap1|Tars|Tesk1|Tgm2|Tor1b|Tor3a|Trib3|Tuba4a|Uba7|Ube2l6|Vars|Wars|Yars
tumor necrosis factor receptor binding(GO:0005164)	5	16	19	31.3	84.2	0	0.0182	Ltb|Tnf|Tnfsf10|Tnfsf13b|Tnfsf15
protein dimerization activity(GO:0046983)	54	655	688	8.2	95.2	0	0.0182	Adora2a|Asns|Atf4|Atf5|Batf|Batf2|Bcl2a1d|Bst2|Cars|Ccl5|Cebpb|Csf1|Daxx|Dck|Ddit3|Ece1|Eno2|Foxp4|Fzd1|Gars|Gca|Gch1|Gsr|Gsta4|Irak2|Irak3|Itgal|Itgb1|Itgb2|Jdp2|Junb|LOC683722|Lcn2|Met|Mlx|Myc|Nampt|Nfe2l1|Nfkb1|Nlrc4|Nos2|Nr1h3|Nr3c1|Plek|Pml|Psmc3ip|Psph|Pvrl2|Slc11a1|Sqstm1|Sycp3|Tap1|Tlr2|Trex1
semaphorin receptor activity(GO:0017154)	3	7	7	42.9	100.0	0.001	0.0327	Nrp1|Nrp2|Plxnd1
glutathione binding(GO:0043295)	4	12	12	33.3	100.0	0.001	0.0327	Gsr|Gsta3|Gsta4|Ptges
kinase binding(GO:0019900)	22	269	283	8.2	95.1	0.001	0.0327	Cd44|Ceacam1|Dlg1|Dusp2|Fas|Itgb1|Itgb2|Jak2|LOC683722|Met|Nek6|Nod2|Pfkl|Plek|Prdx3|Prkcd|Ptk2b|Ptprj|Sdc4|Slc2a1|Sqstm1|Trib3
iron ion transmembrane transporter activity(GO:0005381)	3	7	7	42.9	100.0	0.0015	0.0450	Slc11a2|Slc25a37|Slc39a14
zinc ion transmembrane transporter activity(GO:0005385)	4	15	16	26.7	93.8	0.0015	0.0450	Slc11a2|Slc30a1|Slc39a1|Slc39a14|Slc39a4l
amino acid transmembrane transporter activity(GO:0015171)	9	66	67	13.6	98.5	0.0015	0.0450	Slc13a3|Slc1a4|Slc1a5|Slc3a2|Slc6a9|Slc7a1|Slc7a11|Slc7a2|Slc7a5
magnesium ion binding(GO:0000287)	15	149	167	10.1	89.2	0.0015	0.0450	Atp11b|Atp8b4|Csnk1g3|Dck|Eno2|Farsb|Irak3|Map3k8|Mark1|Mast4|Mthfd2|Nek6|Nt5c1a|Psph|RGD1562091
manganese ion transmembrane transporter activity(GO:0005384)	3	6	6	50.0	100.0	0.002	0.0571	Slc11a1|Slc11a2|Trpm2
tumor necrosis factor binding(GO:0043120)	3	7	7	42.9	100.0	0.002	0.0571	Fas|Ltb|Tnfrsf1b
dicarboxylic acid transmembrane transporter activity(GO:0005310)	4	15	15	26.7	100.0	0.0025	0.0666	Slc13a3|Slc1a4|Slc1a5|Slc25a10
integrin binding(GO:0005178)	7	51	55	13.7	92.7	0.0025	0.0666	Actn1|Icam1|Itga5|Itgb1|Lyn|Mmp14|Ppap2b
alpha-actinin binding(GO:0051393)	3	8	8	37.5	100.0	0.003	0.0759	Adora2a|Itgb1|Pdlim2
deaminase activity(GO:0019239)	4	19	21	21.1	90.5	0.004	0.0956	Adar|Ampd3|Gda|Zbp1
purinergic receptor activity(GO:0035586)	5	31	31	16.1	100.0	0.0045	0.1039	Adora2a|Gpr18|Niacr1|P2ry14|P2ry2
manganese ion binding(GO:0030145)	5	30	31	16.7	96.8	0.006	0.1278	Me1|Pim1|RGD1562091|Slc11a2|Sod2
peptide antigen binding(GO:0042605)	3	10	10	30.0	100.0	0.0065	0.1345	H2-M3|Slc7a5|Tap1
GTP-dependent protein binding(GO:0030742)	3	11	11	27.3	100.0	0.0065	0.1345	Gch1|Mras|Rapgef5
non-membrane spanning protein tyrosine kinase activity(GO:0004715)	4	20	21	20.0	95.2	0.0075	0.1491	Hck|Jak2|Lyn|Ptk2b
glucocorticoid receptor binding(GO:0035259)	3	12	12	25.0	100.0	0.008	0.1566	Cebpb|Ets2|Psmc3ip
cell adhesion molecule binding(GO:0050839)	6	48	51	12.5	94.1	0.009	0.1708	Itga5|Itgal|Itgb2|PVR|Ptprm|Pvrl2
antioxidant activity(GO:0016209)	6	50	56	12.0	89.3	0.009	0.1708	Gsr|Prdx3|Ptgs2|Sep15|Sod2|Txnrd1
NADP binding(GO:0050661)	5	36	43	13.9	83.7	0.012	0.2125	Gsr|Hsd11b1|Me1|Nos2|Txnrd1
tRNA binding(GO:0000049)	4	22	23	18.2	95.7	0.014	0.2377	Aars|Cars|Xpot|Yars
solute(GO:0015295)	3	15	15	20.0	100.0	0.014	0.2377	Slc11a2|Slc15a3|Slc2a1
receptor regulator activity(GO:0030545)	4	26	26	15.4	100.0	0.0175	0.2879	Ccl5|Il1rn|Sfrp2|Sra1
nucleotide receptor activity(GO:0016502)	4	27	27	14.8	100.0	0.0175	0.2879	Gpr18|Niacr1|P2ry14|P2ry2
oxidoreductase activity, acting on a sulfur group of donors(GO:0016667)	5	44	51	11.4	86.3	0.022	0.3462	Ero1l|Gsr|Pdia5|Ptges2|Txnrd1
oxidoreductase activity, acting on single donors with incorporation of molecular oxygen, incorporation of two atoms of oxygen(GO:0016702)	5	43	44	11.6	97.7	0.0225	0.3508	Egln3|P4ha2|Phyhd1|Pir|Ptgs2
cytokine receptor activity(GO:0004896)	5	41	46	12.2	89.1	0.0235	0.3607	Csf2rb|Ebi3|Il1rap|Il2rg|Il7r
kinase inhibitor activity(GO:0019210)	4	28	28	14.3	100.0	0.0295	0.4149	LOC683722|Pkia|Rhoh|Trib3
fibronectin binding(GO:0001968)	3	18	20	16.7	90.0	0.0295	0.4149	Itgb1|Mmp9|Sdc4
GTPase activity(GO:0003924)	11	138	153	8.0	90.2	0.0395	0.4312	Gbp2|Gbp4|Gbp5|Igtp|Mras|Mx1|Mx2|Rac2|Rhoq|Rras|Tuba4a
peptidase activator activity(GO:0016504)	3	21	21	14.3	100.0	0.0405	0.4358	Mmp14|Psme2|Sfrp2
L-ascorbic acid binding(GO:0031418)	3	20	20	15.0	100.0	0.0435	0.4454	Egln3|Ogfod1|P4ha2
threonine-type peptidase activity(GO:0070003)	3	22	24	13.6	91.7	0.0475	0.4789	Psmb10|Psmb8|Psmb9

Rat microglia; **GO Name - Cellular Component**	Number of genes differentially expressed that were in this pathway/category	Number of genes in this pathway that were on the chip	Number of genes in this category, total	Percent Changed	Percent Present	P-value	Adjusted P-value	Genes that were differentially expressed *and* are in this category
membrane raft(GO:0045121)	21	182	185	11.5	98.4	0	0.0182	Angpt1|Birc2|Birc3|Casp8|Cd14|Dlg1|Efhd2|Fas|Hck|Itgb1|Itgb2|Jak2|Lyn|Myof|Ptgs2|Ptk2b|Rhoq|Sdc4|Slc2a1|Tnf|Tnfrsf1b
extracellular space(GO:0005615)	48	585	632	8.2	92.6	0	0.0182	Angpt1|C3|Camp|Ccl12|Ccl20|Ccl3|Ccl4|Ccl5|Ccl7|Ccl9|Cd14|Chi3l1|Cklf|Csf1|Csf2|Cxcl1|Cxcl10|Cxcl13|Cxcl16|Cxcl2|Cxcl3|Cxcl9|Es1|Gdf15|Havcr1|Icam1|Il10|Il18bp|Il1a|Il1b|Il1rn|Il6|Itga5|Kng1|LOC360228|Lcn2|Ltb|Met|Mmp9|Nppb|Retn|Serpinb2|Sfrp2|Srgn|Tnf|Tnfsf10|Tnfsf15|Vash1
I-kappaB/NF-kappaB complex(GO:0033256)	4	5	5	80.0	100.0	0	0.0182	Bcl3|Nfkb2|Nfkbia|Nfkbib
external side of plasma membrane(GO:0009897)	27	169	179	16.0	94.4	0	0.0182	Cd274|Cd40|Cd44|Cd69|Cxcl10|Cxcl9|Ece1|Emr1|Fas|Fcgr2b|Gsr|H2-M3|Icam1|Icoslg|Il2rg|Il6|Il7r|Itga5|Itgal|Itgax|Klrk1|Ly75|Tlr2|Tmem123|Tnf|Tnfrsf14|Tnfsf13
CD95 death-inducing signaling complex(GO:0031265)	3	4	4	75.0	100.0	0	0.0182	Casp8|Cflar|Fas
phagocytic vesicle membrane(GO:0030670)	4	8	8	50.0	100.0	0	0.0182	Anxa3|Irgm|Rab11fip1|Slc11a1
immunological synapse(GO:0001772)	5	14	14	35.7	100.0	0	0.0182	Dlg1|Icam1|Itgal|Ptprj|Rhoh
cytosol(GO:0005829)	97	1319	1373	7.4	96.1	0	0.0182	Acot7|Actn1|Actn4|Arhgef3|Asns|Bcat1|Birc2|Cars|Casp7|Casp8|Ch25h|Chac1|Clic4|Daxx|Dck|Dlg1|Eif2s2|Eif4ebp1|Eprs|Farsb|Fgr|Fyb|G1p2|Garnl4|Gars|Gbp4|Gch1|Gk|Gsr|Gsta3|Hebp1|Iars|Ifih1|Igtp|Ikbke|Il1a|Irak2|Irf7|Jak2|LOC683722|Lars|Lcn2|Lims1|Lpin2|Lyn|Map3k8|Mapkapk2|Me1|Mgrn1|Mocs2|Nampt|Nars|Ncf1|Ncf4|Nfkb1|Nfkb2|Nfkbia|Nfkbib|Nod2|Nos2|Nr3c1|Pde4b|Pfkl|Pftk1|Pik3cg|Pik3r5|Plek|Prex1|Prkcd|Prmt3|Pstpip2|Ptges2|Ptpn1|Ptpn12|RGD1308251|Rac2|Ralgds|Rilpl2|Rnd1|Rras|Sars|Slc7a5|Spry1|Sqstm1|Stard5|Stat1|Stat2|Tank|Tars|Tesk1|Tuba4a|Txnrd1|Uba7|Ube2l6|Usp18|Vars|Vasp
cell surface(GO:0009986)	30	294	305	10.2	96.4	0	0.0182	Adamts7|Cd14|Cd38|Cd44|Clic4|Fas|Fut4|Fzd1|Havcr1|Hspa9|Icam1|Il1a|Il2rg|Irak2|Itgal|Itgb1|Itgb2|Nod2|Nrp1|PVR|Pdlim2|Ptprj|Pvrl2|Sdc1|Sdc4|Slc11a1|Slc11a2|Slc3a2|Tlr2|Tnf
cytoplasm(GO:0005737)	205	3465	3657	5.9	94.7	0.001	0.0327	Aars|Acot7|Actn1|Actn4|Adar|Anxa3|Apbb3|Apobec3f|Aqp12b|Ard1a|Arhgap27|Arl6ip5|Ass1|Atf4|Atf5|Bcat1|Bcl3|Birc3|Camkk2|Camp|Cars|Casp7|Casp8|Ccl5|Cd44|Cdc42ep2|Cdr2|Cebpb|Cflar|Cib1|Ckap2|Clic2|Clic4|Coro1b|Csnk1g3|Daxx|Ddit3|Dhx58|Diaph2|Dtx3l|Dusp16|Egln3|Eif2b3|Eif4ebp1|Eno2|Eprs|Fam129a|Farsb|Foxp4|Ftsjd2|Gars|Gca|Gch1|Gk|Gsr|Gsta3|Hcls1|Hebp1|Hmgn3|Hn1l|Hsd11b1|Iars|Ifi44|Ifih1|Ifit1|Ikbke|Il1rn|Irak3|Itga5|Jak2|LOC683722|Lars|Lgals9|Lst1|Lyn|Map3k8|Mapk6|Mapkapk2|Mark1|Mars|Me1|Mefv|Met|Mgrn1|Mlx|Mmp14|Mocs2|Mvp|Mx1|Mx2|Myc|Nadk|Naip2|Nampt|Nars|Ncf1|Ncf4|Nek6|Nfkb1|Nfkb2|Nfkbia|Nfkbib|Nfkbie|Nlrp3|Nmi|Nos2|Nostrin|Nppb|Nqo1|Nr1h3|Nr3c1|Nt5c1a|Ntn1|Nup62|Ogfr|Optn|Parp14|Parp9|Pde4b|Pdlim2|Pfkl|Pftk1|Pik3ap1|Pim1|Pinx1|Pir|Pitpnm1|Piwil2|Pkia|Pla2g16|Plagl2|Plek|Pml|Polr1e|Prdx3|Prkcd|Prkch|Prmt3|Psmb10|Psmb8|Psmb9|Ptges|Ptgs2|Ptk2b|Ptpn12|Ptprm|Pycs|RGD1305685|RGD1308251|Rab11fip1|Rac2|Rai14|Rbpj|Rhoh|Rhoq|Rilpl2|Ripk3|Rtp3|Rtp4|Sars|Scin|Sdc1|Sept11|Serpinb2|Sh3bp1|Skil|Sla|Slc11a2|Slc25a24|Slc2a1|Slc7a2|Slc7a5|Snx20|Socs3|Sod2|Sp140|Sqstm1|Sra1|Ssu72|Stat1|Stat2|Tars|Tbc1d2|Tgm2|Tlr2|Tlr7|Tnip1|Tor1b|Tor3a|Trim21|Trim26|Trim35|Tuba4a|Txnrd1|Ubd|Upp1|Vars|Vash1|Vasp|Wars|Xpot|Yars|Zc3h12a|Zc3hav1|Zfand2a
plasma membrane(GO:0005886)	133	2119	2283	6.3	92.8	0.0015	0.0450	Acsl1|Adora2a|Agtrap|Angpt1|Anxa3|Atf4|Atp11b|Bst1|Bst2|Cd14|Cd200|Cd274|Cd300lf|Cd38|Cd40|Cd44|Cd97|Cdc42ep2|Cdh5|Ceacam1|Chic2|Cib1|Cish|Clic4|Cpd|Csf1|Csf2rb|Dlg1|Ece1|Efnb2|Emr1|Eno2|F10|F2rl2|Fam38a|Fas|Fcgr2b|Fcgr3a|Fgr|Fzd1|Gca|Gcgr|Gnb5|Gpr18|Gpr68|Gprc5d|H2-T23|Hcls1|Icam1|Ifitm3|Il1rap|Il2rg|Irak2|Irgm|Itga5|Itgal|Itgb1|Itgb2|Jag1|Kcnj1|Kcnj2|Ly6b|Lyn|Met|Mgrn1|Mras|Myof|Nab1|Ncf1|Neu1|Nfkbia|Niacr1|Nod2|Nostrin|Nrp1|Nrp2|P2ry14|P2ry2|PVR|Pak1ip1|Parp14|Pftk1|Pik3ap1|Pla2g16|Plxnd1|Ppap2b|Prex1|Prkcd|Prkch|Ptger2|Ptger4|Ptk2b|Ptprj|Pvrl2|Rhoh|Rhoq|Rilpl2|S1pr2|Sdc4|Sema4a|Sema4d|Sgms2|Slamf7|Slc11a1|Slc11a2|Slc13a3|Slc16a3|Slc24a6|Slc2a1|Slc30a1|Slc39a1|Slc39a14|Slc39a4l|Slc3a2|Slc7a1|Slc7a11|Slc7a2|Slc7a5|Snx20|Spry1|Stat2|Steap3|Stx3|Tgm2|Tlr2|Tlr7|Tnf|Tnfrsf14|Tnfsf15|Trpm2|Vamp5|Vasp|Vom2r41|Zc3h12a
integrin complex(GO:0008305)	5	23	26	21.7	88.5	0.002	0.0571	Itga5|Itgax|Itgb1|Itgb2|Lyn
specific granule(GO:0042581)	3	9	10	33.3	90.0	0.003	0.0759	Anxa3|Camp|Stx3
recycling endosome(GO:0055037)	5	30	32	16.7	93.8	0.0035	0.0862	Lmtk2|Rab11fip1|Slc11a2|Tnf|Vamp8
focal adhesion(GO:0005925)	9	83	85	10.8	97.6	0.004	0.0956	Actn1|Itga5|Itgb1|Lims1|Pdlim2|Ptk2b|Sdc1|Sdc4|Vasp
MHC protein complex(GO:0042611)	5	33	52	15.2	63.5	0.008	0.1566	H2-M3|RT1-CE13|RT1-M6-1|RT1-N1|RT1-T24-1
endomembrane system(GO:0012505)	6	45	45	13.3	100.0	0.0085	0.1638	Adora2a|Cdc42ep2|Jak2|Slc11a2|Vamp5|Vamp8
leading edge membrane(GO:0031256)	6	48	49	12.5	98.0	0.009	0.1708	Adora2a|Atf4|Itga5|Itgb1|Myo1d|Plek
proteasome core complex(GO:0005839)	3	14	14	21.4	100.0	0.0135	0.2318	Psmb10|Psmb8|Psmb9
lysosomal membrane(GO:0005765)	6	62	65	9.7	95.4	0.032	0.4267	Acp2|Acpp|Neu1|Slc11a2|Slc15a3|Vamp8
stress fiber(GO:0001725)	4	33	36	12.1	91.7	0.042	0.4386	Actn1|Actn4|Pdlim2|Sept11

Supplementary Table 3: Sequence of primers used for RT Quantitative PCR.

RT QPCR primers	Forward Primer	Reverse Primer
T-bet	GGACGATCATCTGGGTCACATTGT	GCCAGGGAACCGCTTATATG
Gata-3	CTGACGGAAGAGGTGGACGT	GTGGTTGCCTTGACCATCG
Rorc	AGCAGTGTAATGTGGCCTAC	GCACTTCTGCATGTAGACTG
Foxp3	AGAGCCCTCACAACCAGCTA	CCAGATGTTGTGGGTGAGTG
IFN-γ	TCAAGTGGCATAGATGTGGAAGAA	TGGCTCTGCAGGATTTTCATG
IL-4	ACAGGAGAAGGGACGCCAT	GAAGCCCTACAGACGAGCTCA
IL-17F	GAGGATAACACTGTGAGAGTTGAC	GAGTTCATGGTGCTGTCTTCC
IL-12p35	CACGCTACCTCCTCTTTTTG	CAGCAGTGCAGGAATAATGTT
IL-12p40	TCCCCATTCCTACTTCTCCCTC	GGAACGCACCTTTCTGGTTACA
IL-23p19	CCAGCGGGACATATGAATCTACT	CTTGTGGGTCACAACCATCTTC
MHC II	CCAAGGTGGACTGCAAAACATAG	CACAAAGCAGATGAGGGTGTTG
CD86	TCAAAACATAAGCCTGAGTGAGC	CAGGTACTTGGCATTCACACTATC
CXCL10	CCAAGTGCTGCCGTCATTTTC	GGCTCGCAGGGATGATTTCAA
CCL2	TGGCTCAGCCAGATGCAGT	TTGGGATCATCTTGCTGGTG
GAPDH	CAAGGCCGAGAATGGGAAG	GGCCTCACCCCATTTGATGT.
